# Tracking hidden crisis in India’s capital from space: implications of unsustainable groundwater use

**DOI:** 10.1038/s41598-021-04193-9

**Published:** 2022-01-13

**Authors:** Shagun Garg, Mahdi Motagh, J. Indu, Vamshi Karanam

**Affiliations:** 1 Department of Civil Engineering, Indian Institute of Technology Bombay, Powai, Mumbai, India 400076; 2 Institute for Photogrammetry and Geo-Information, Leibniz University Hannover, Hannover, Germany 30167; 3 Remote Sensing and Geoinformatics, GFZ German Research Centre for Geosciences, Potsdam, Germany 14473; 4 Interdisciplinary Centre for Climate Studies, Indian Institute of Technology Bombay, Powai, Mumbai, India 400076; 5 Southern Methodist University, Dallas, TX, USA; 6 Present Address: Future Infrastructure and Built Environment (FIBE), Department of Engineering, University of Cambridge, Cambridge, CB2 1PZ UK

**Keywords:** Natural hazards, Sustainability

## Abstract

National Capital Region (NCR, Delhi) in India is one of the fastest-growing metropolitan cities which is facing a severe water crisis due to increasing water demand. The over-extraction of groundwater, particularly from its unconsolidated alluvial deposits makes the region prone to subsidence. In this study, we investigated the effects of plummeting groundwater levels on land surface elevations in Delhi NCR using Sentinel-1 datasets acquired during the years 2014–2020. Our analysis reveals two distinct subsidence features in the study area with rates exceeding 11 cm/year in Kapashera—an urban village near IGI airport Delhi, and 3 cm/year in Faridabad throughout the study period. The subsidence in these two areas are accelerating and follows the depleting groundwater trend. The third region, Dwarka shows a shift from subsidence to uplift during the years which can be attributed to the strict government policies to regulate groundwater use and incentivizing rainwater harvesting. Further analysis using a classified risk map based on hazard risk and vulnerability approach highlights an approximate area of 100 square kilometers to be subjected to the highest risk level of ground movement, demanding urgent attention. The findings of this study are highly relevant for government agencies to formulate new policies against the over-exploitation of groundwater and to facilitate a sustainable and resilient groundwater management system in Delhi NCR.

## Introduction

Land Subsidence (LS) is considered as one of the severe, often overlooked, geological hazards, which is causing more and more damage every year^1^. It can be attributed to anthropogenic activities like underground extraction of natural resources^2^, minerals^3^, oil and gas^4^, water^5^, as well as natural events such as soil compaction^6^, earthquake^7^, and loess deposits^8^. According to U.S. Geological Survey (USGS), more than 80% of land subsidence across the world is caused due to excessive groundwater extraction^9^. Groundwater extraction from the aquifer system increases the effective intergranular stress and causes rearrangement of soil particles resulting in aquifer compaction and eventual land subsidence^10^. Well-known case studies in Mexico^11–13^, Iran^14–18^, China^19–21^, and India^22,23^ indicate a strong correlation between LS and groundwater extraction.

The growing population expansion and urbanization require an enormous amount of water to fulfill the demand. During the past decade, groundwater extraction rate has increased drastically to about 145 cubic kilometers per year^24^ posing a huge threat of LS, particularly in the areas dominated by alluvial soil. With direct and indirect consequences, LS can result in structural damage of roads, buildings, dikes, railways, etc. as well as cause flood expansion, reduced aquifer capacity, gradient change of water mains, and sewage pipes, affecting critical underground infrastructure^25^. The cost of damage caused by land subsidence in 2012 alone is estimated to exceed billions of dollars^1^.

Measuring and understanding the spatio-temporal extent of LS is crucial to mitigate its hazards. Conventional methods to monitor LS, such as Continuous Global Positioning System (GPS) measurements, leveling, extensometers are either laborious or expensive. A widely known and powerful method to monitor subsidence is Interferometric Synthetic Aperture Radar (InSAR)^26^. The InSAR technique exploits information from microwave sensors onboard satellites to identify minute variations in earth’s surface. InSAR has been extensively used to monitor surface deformation in many parts of the world, including, but not limited to Mexico city^12,13,27–30^, Iran^10,14–16,31,32^, Vietnam^33,34^, Indonesia^35–37^, Spain^38,39^, Tokyo^40^, Beijing^5,20^, Houston^41,42^, Italy^43,44^, and Kolkata^22,23,45^. InSAR also offers useful information about the effect of subsidence on urban areas^43^, infrastructure^20^, bridges^46–49^, airports^10,20,50^, and other facilities. Recently, many organizations have shown interest in accessing the associated risk along with the spatio-temporal distribution of subsidence. For instance, Torres et al.^51^ and Goense^52^ used population density and subsidence horizontal gradient to perform risk zoning in Mexico city using hazard vulnerability and risk approach. A recent study predicts that 19% of the population worldwide is likely to face subsidence in near future and India ranks first in terms of exposed population and spatial extent of population^53^.

While many Indian cities lying in the fluvial Indo-Gangetic Plain are prone to land subsidence, not many cities are known to subside. Delhi National Capital Region (NCR), for instance, is one of the fastest-growing metropolitan cities, with a population density of nearly 30,000 people per square mile^54^. It is predicted that Delhi will become the world’s most populous city, with 37.2 million people by 2028^55^. The reason for this unprecedented rise in population can be attributed to the booming economy and highest per capita income of Delhi NCR which attracts migrants from different parts of the country^56^. As a result of this expansion, NCR has been experiencing an enormous water demand. According to the 2017 Economic Survey of India^57^, around 6,25,000 households (18% population) do not have access to pipe water supply. They rely either on groundwater or on private tankers for their daily needs. The difference between demand and supply of water is more than 750 million liters a day^58^. At certain locations in southwest Delhi, groundwater table is available at a depth of 80 m below ground and this continues to deplete at the rate of 3–4 m/year. The Central Ground Water Authority (CGWA) India in December 2018, introduced ‘Water Conservation Fees (WCF)’ to groundwater extraction for domestic and industrial purposes. The penalty varies depending on the amount of extraction and the exploited zone^59^ (highest for overexploited, critical, and semi-critical blocks). However, it exempts individual households and agricultural users (the largest user of groundwater). Moreover, the current policies are solely based to mitigate the water scarcity problem in NCR and do not take into account the hazardous effect of land subsidence. The reason for this is that LS is often overlooked in India; There are only a handful of studies conducted to address this issue^60–64^, most of which are limited to coal mining. The current water crisis in India, particularly in northern states^65,66^, poses a huge threat and raises an urgency to monitor Land Subsidence^53^.

**Figure 1:**
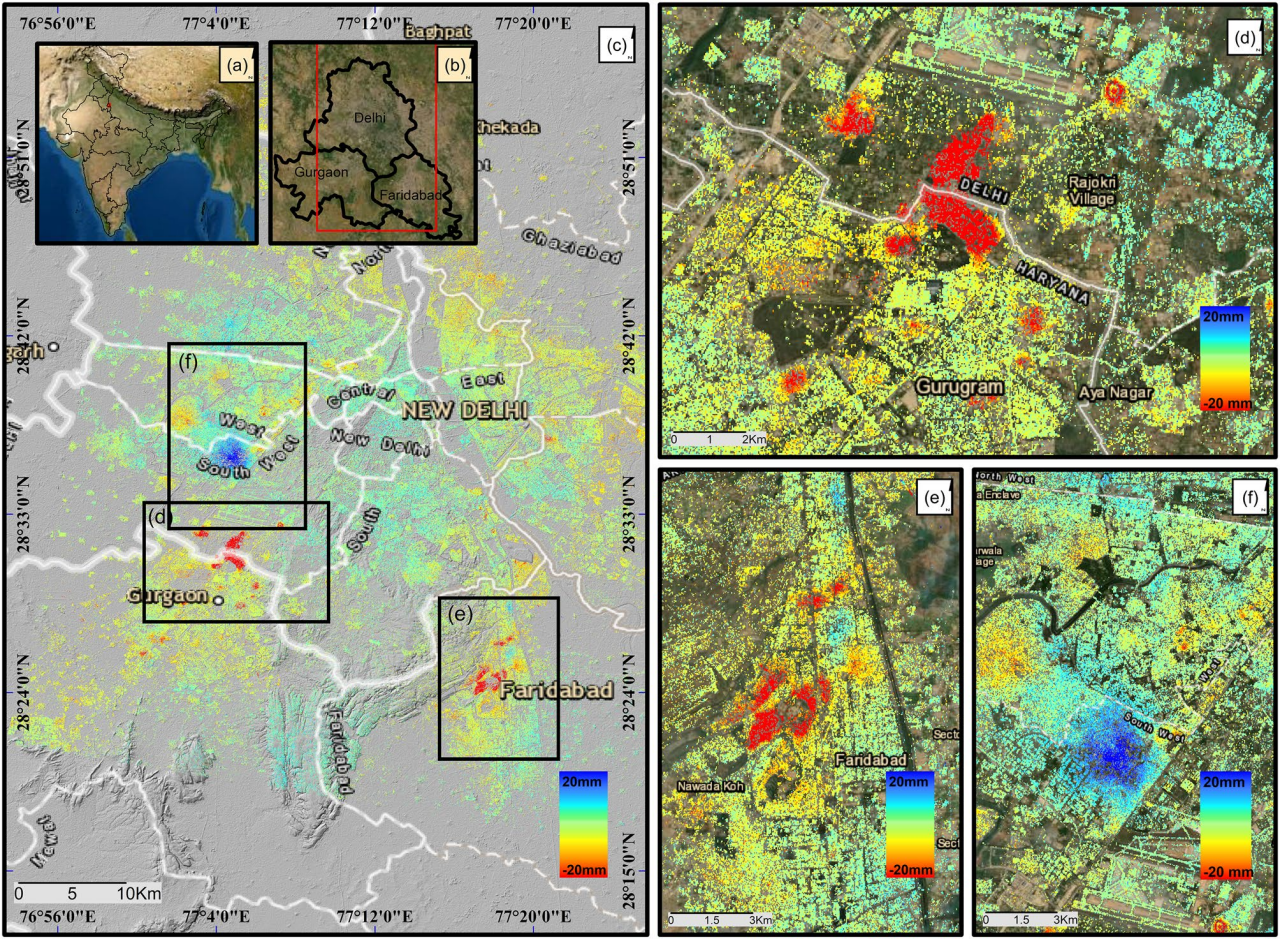
(**a**) and (**b**) shows the administrative boundaries of India and National Capital Region (NCR) of India which includes Delhi, and neighboring urban cities. In this study we focused on Delhi, Faridabad and Gurgaon. (**c**) shows the deformation results (in line of sight) obtained during phase 2 (2016–2018) analysis acquired in ascending direction. The color bar represents the land subsidence velocity in (mm/year). Red color represents high subsidence region, whereas blue color represents an uplift. Three displacement features (two of them undergoing subsidence, and one showing uplift) are delineated and are marked with a black square in (**c**). The close-up view of the three areas is shown in (**d**), (**e**), (**f**) respectively. Here (**d**) refers to Kapashera; (**e**) refers to Faridabad, and (**f**) refers to Dwarka. Maps were generated in ArcMap software (Version 10.4; Base Map: World Map; copyright and licensed by ESRI https://desktop.arcgis.com/).

Here, for the first time, we present an extensive study of land subsidence in Delhi NCR (Fig. 1b) derived using freely available sentinel-1 datasets acquired between 2014 and 2020 in ascending and descending directions. We applied persistent scatterer interferometry (PSI)^67^ using Stanford Method for Permanent Scatterers (StaMPS)^68^ to derive the long-term time series subsidence results. The overall time-period is split into three phases to avoid temporal decorrelation; Phase 1 (2014–2016), phase 2 (2016–2018), and phase 3 (2018–2019). Individual time series are then merged to produce a long-term displacement time series. A comparative evaluation of groundwater and land subsidence suggests that the local excessive groundwater pumping is the major cause of land subsidence. To identify the risk of ground movement in the study area, a risk map is derived using seven representative indices employing hazard vulnerability and risk approach. The risk map is further classified into three risk classes viz, high, medium, and low risk. The findings of this study can be used by Delhi Jal Board (DJB) and the Government of Delhi to formulate new policies against the over-exploitation of groundwater, especially in areas under high threat of subsidence, and to facilitate a sustainable and resilient groundwater management system.

## Results

Figure 1 shows the 2016–2018 ground deformation velocity (in line of sight) in the Delhi NCR region. The results are produced from time series analysis of Sentinel-1 SAR images acquired in ascending orbit from August 2016 to September 2018. Three displacement features (two of them undergoing subsidence, and one showing uplift) are delineated with a black square in Fig. 1c. A closer view of the three regions is shown in Fig. 1d–f. These are (d) Kapashera (e) Faridabad, and (f) Dwarka. The subsidence mainly occurs in urban areas with a high population density. A detailed investigation of each area from October 2014-January 2020 is presented in the following sections.

### Continuous ground deformation in Kapashera (Southwest-Delhi, Gurgaon border)

**Figure 2:**
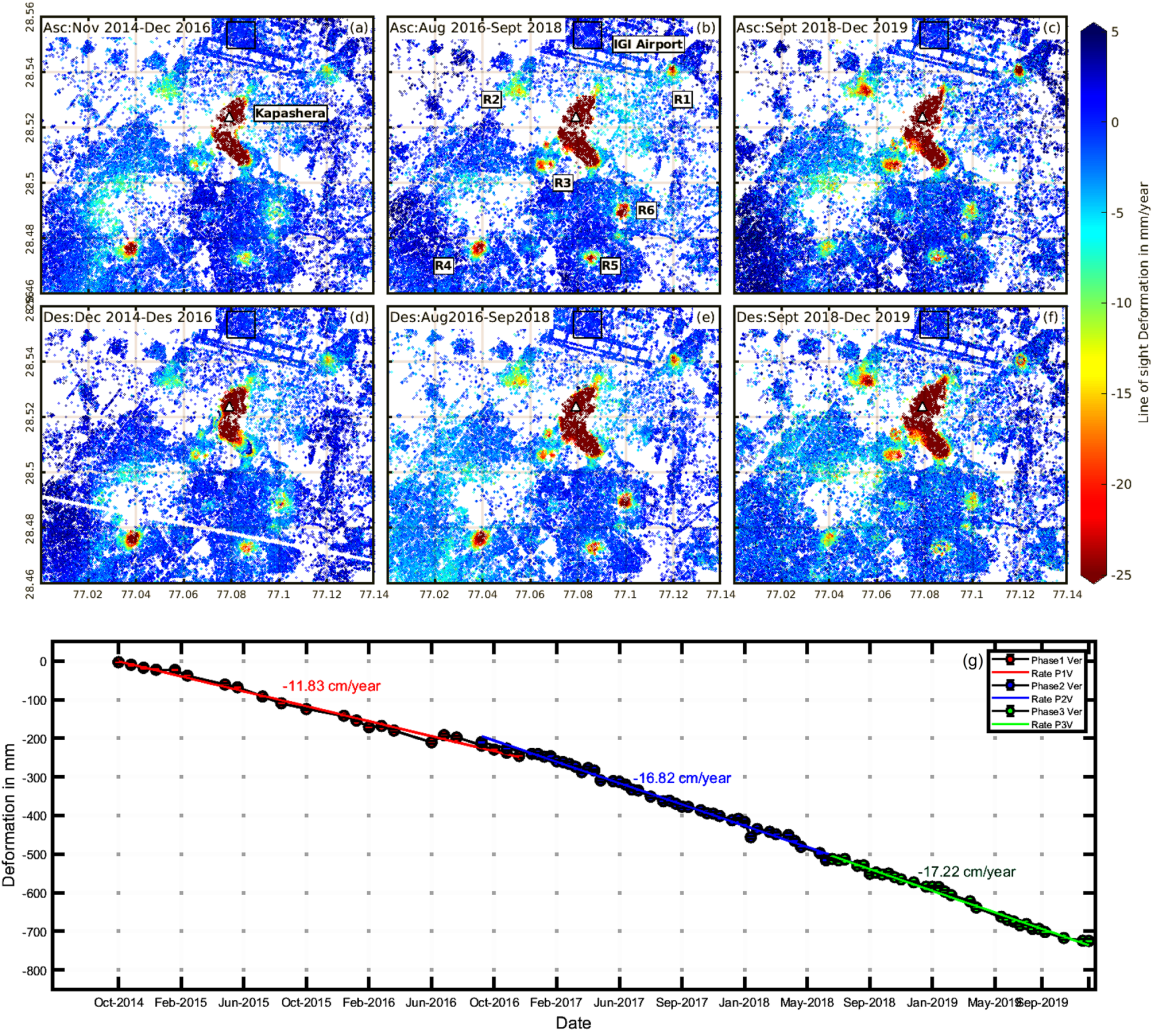
The Line-of-sight velocity plots from ascending and descending datasets for Kapashera (Fig. 1d). The first-row plots (**a**–**c**) are the results from ascending datasets while the bottom row (**d**–**f**) is the subsidence velocity plots from descending datasets. The three plots in each row are the subsidence rate estimated during phase 1 (2014–2016), phase 2 (2016–2018), and phase 3 (2018–2019) respectively. The black square at the IGI airport in (**b**) is chosen as a reference area. Red color depicts subsidence velocity in mm/year whereas blue shows no deformation. In (**b**) R1–R6 are small subsiding regions near Kapashera. The region R1 is Mahipalpur village, Delhi; R2 is Bijwasan Harijan Basti; R3 is sector 22A Gurgaon; R4 is the Sanjay gram colony; R5 is chack Sadhu, Delhi and R6 is Nathupur, Delhi. (**g**) shows the vertical time series of the region marked with white triangle in (**a**–**c**). The red, blue and green color represents the vertical time series of deformation observed during phase 1, phase 2, and phase 3 respectively and are derived using ascending datasets. The subsidence rate is estimated using simple linear regression.

The largest subsidence feature is a slum settlement in south-west Delhi (Fig. 1d) which shares its borders with Gurgaon, Haryana. It lies in the neighborhood of Udyog Vihar and Indra Gandhi International (IGI) airport and covers an approximate area of 12.5 km^2^. The average subsidence velocity in the line-of-sight direction derived using sentinel-1 ascending and descending datasets during 2014–2016 (phase 1), 2016–2018 (phase 2), and 2018 -2019 (phase 3) are illustrated in Fig. 2a–f. An inter-comparison of sentinel-1 ascending and descending pass results shows a similar pattern. Figure 2g shows the vertical time series derived using ascending data and average subsidence velocity of the points marked with white triangle in Fig. 2a–c; The rate of subsidence follows a strong continuous rising trend throughout the study period. During the years 2014–2016, the subsidence velocity is found to be approximately 11 cm/year which rose significantly by almost 50% over the next two years to around 17 cm/year. The trend remained almost same during 2018–2019. In addition to the main subsidence feature, there also exists a few slow subsidence zones which are expanding with time. These regions are marked as R1–R6 in Fig. 2b. The region R1 is Mahipalpur village, Delhi; R2 is Bijwasan Harijan Basti; R3 is sector 22A Gurgaon; R4 is the Sanjay gram colony; R5 is chack Sadhu, Delhi and R6 is Nathupur, Delhi. Most of these areas are subsiding between 15–40 mm/year. A comparison of the velocity plots from the three phases shows that the subsidence feature is rapidly expanding towards the IGI Airport Delhi, causing a threat to the structure. It is, therefore, crucial to continue monitoring subsidence in the area and take appropriate steps to mitigate potential damage to nearby airport.

### Subsidence—uplift trend in Dwarka, Delhi

**Figure 3:**
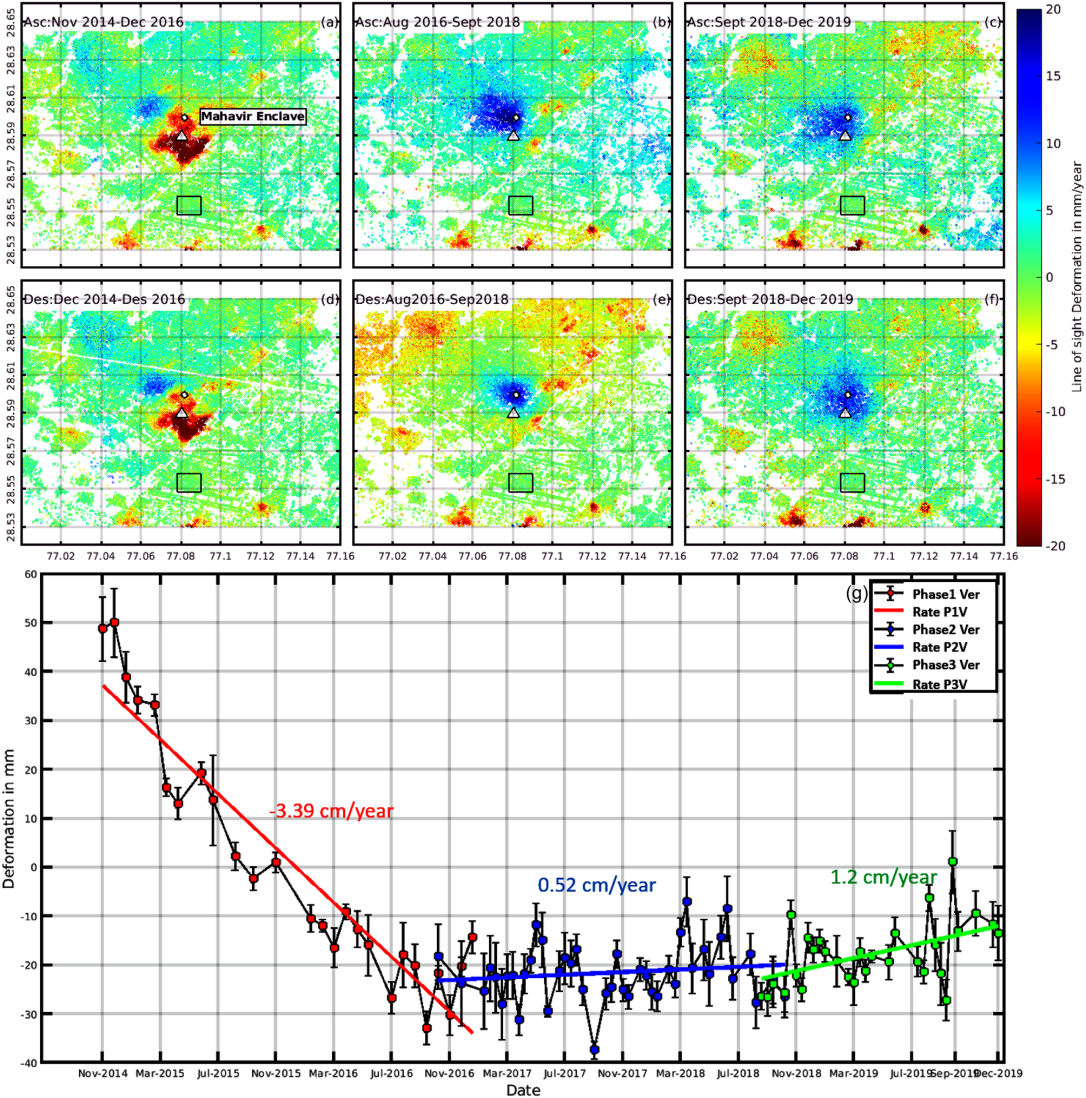
The Subsidence velocity plots from ascending and descending datasets for Dwarka (Fig. 1f). The first-row plots (**a**–**c**) are the results from ascending datasets while the bottom row (**d**–**f**) is the subsidence velocity plots from descending datasets. The three plots in each row are the subsidence rate estimated during phase 1 (2014–2016), phase 2 (2016–2018), and phase 3 (2018–2019) respectively. The black square at the IGI airport marked with black box is chosen as a reference area. Red color depicts subsidence velocity in mm/year whereas blue shows uplift. (**g**) shows the vertical time series of the region marked with white triangle in (**a**–**f**). The red, blue and green color represents the vertical time series observed during phase 1, phase 2, and phase 3 respectively and are derived using ascending datasets. The subsidence rate is estimated using simple linear regression.

The second significant displacement feature in the study area is Dwarka (Fig. 1f). It is the largest suburb in Asia and serves as the administrative head office of South West Delhi^69^. A comparison of the velocity map from the three-time periods in Fig. 3a–f, shows an uplift trend. During Phase 1 (2014–2016), the region was undergoing subsidence with an approximate rate of 3.5 cm/year, which has been shifted, into a gradual uplift in the subsequent phases. The rate of uplift in phase 2 (2016–2018) and phase 3 (2018–2020) is estimated at around 0.5 and 1.2 cm/year, respectively. The subsidence map derived from ascending (Fig. 3a–c) and descending pass (Fig. 3d–f) shows a good agreement, indicating the reliability of the results. Figure 3g shows the vertical time series derived using ascending data of the points marked with white triangle in Fig. 3a–c.

The main reason for this change in the behavior of subsidence pattern can be associated with the swelling behavior of soil due to the rise in groundwater table and the consequent reduction of effective stress in the soil. Delhi government introduced several strict policies to improve the groundwater condition in the area. For example, Delhi Jal Board (DJB) in July 2016, made it compulsory to install a rainwater harvesting system for the owners of property more than 500 square meters to promote rainwater as an alternative to groundwater and penalize illegal pumping by imposing heavy fines^70^. In addition to this, residents of Dwarka revived a 200-year-old water body named ‘naya jhod’ of 10 million storage capacity in august 2015, which not only reduced water demand but also acted as a very good source of groundwater recharge^71^. Wang et al.^72^ discussed that if the process of land subsidence due to long-term groundwater extraction is followed by groundwater recovery, then the relevant soil layer may rebound and the ground surface may rise. Phien-Wej et al.^73^ conducted field experiment by artificially recharging an aquifer in Bangkok and recorded a land rebound of 3 mm. This strengthens our hypothesis that groundwater recharge could be the major reason for the uplift in region. However detailed hydrogeological analysis is required to confirm the hypothesis.

### Accelerating rate of land subsidence in Faridabad, Haryana

**Figure 4:**
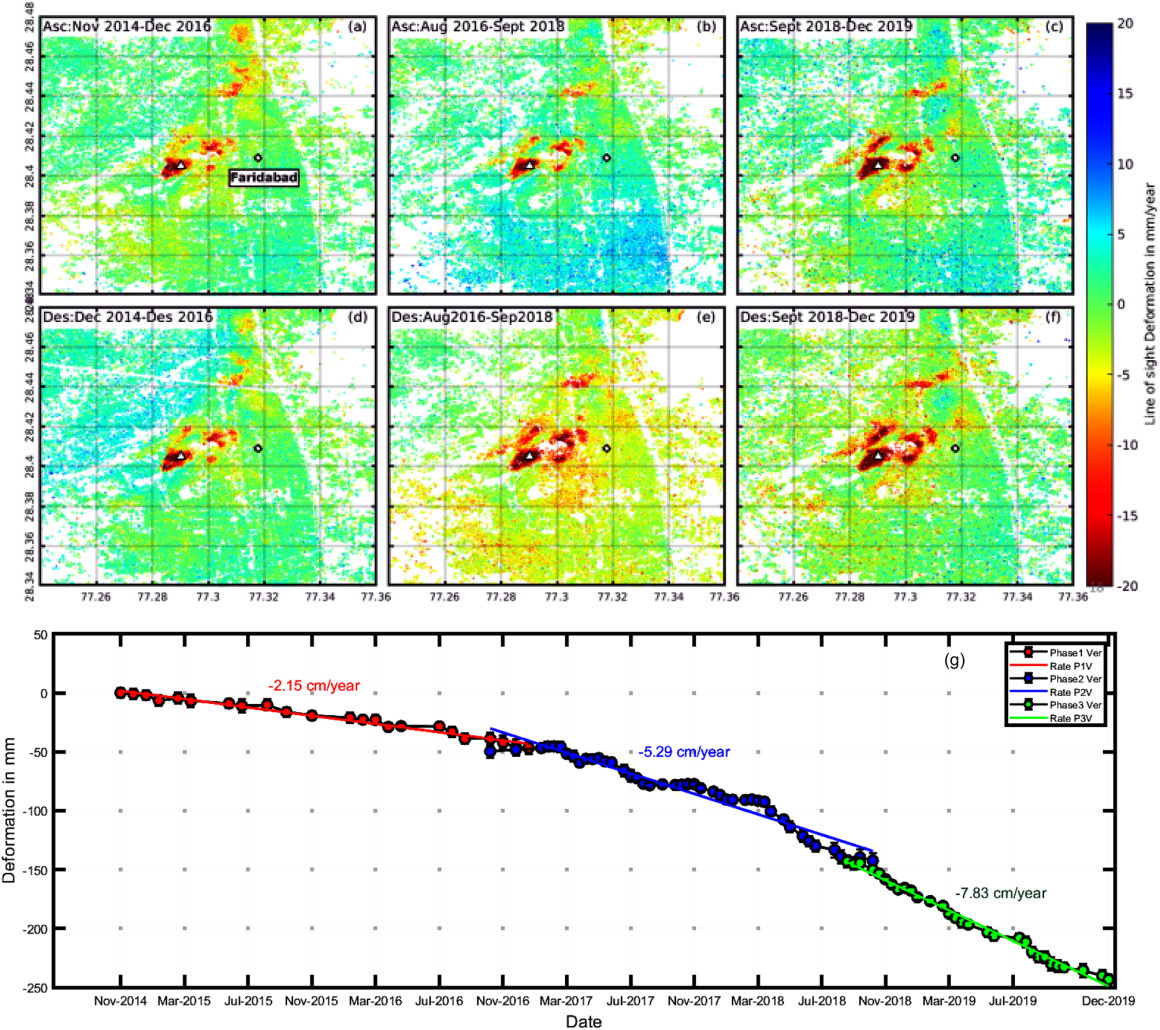
The Subsidence velocity plots from ascending and descending datasets for Faridabad (Fig. 1e). The first-row plots (**a**–**c**) are the results from ascending datasets while the bottom row (**d**–**f**) is the subsidence velocity plots from descending datasets. The three plots in each row are the subsidence rate estimated during phase 1 (2014–2016), phase 2 (2016–2018), and phase 3 (2018–2019) respectively. Red color depicts subsidence velocity in mm/year whereas blue shows uplift. (**g**) shows the vertical time series of the region marked with white triangle in (**a**–**f**). The red, blue, and green color represents the vertical time series observed during phase 1, phase 2, and phase 3 respectively and are derived using ascending datasets. The subsidence rate is estimated using simple linear regression.

The next subsidence feature in Delhi NCR was observed to occur in Faridabad, Haryana (Fig. 1e). Faridabad is the industrial capital of Haryana and is one of the fastest-growing cities in the world. The subsidence velocity derived using ascending and descending pass of sentinel-1 in Fig. 4 are in general agreement for all three-time periods. The time series in Fig. 4g illustrates the accelerating rate of subsidence throughout the study period. From 2014 to 2016, the maximum subsidence rate was relatively low, around 2.15 cm/year and the spatial extent was small. However, during subsequent years, the deformation rates surged dramatically, to an approximate rate of 5.3 cm/year by the end of 2018 and 7.83 cm/year for the year 2018–2019. The results also indicate that the extent of subsidence increased continuously with time. The deeper groundwater levels, and the high rate of groundwater extraction (discussed in subsequent sections) in Faridabad, explain the high extent of subsidence in the region. The primary reason for the plummeting groundwater levels is illegal pumping; More than 100,000 of the connections in Faridabad are illegal and are being used for almost a decade^74^. The area which is affected the most is found to be New Industrial Town (NIT) Faridabad.

Discussion

### Comparative evaluation of groundwater and land subsidence

**Figure 5:**
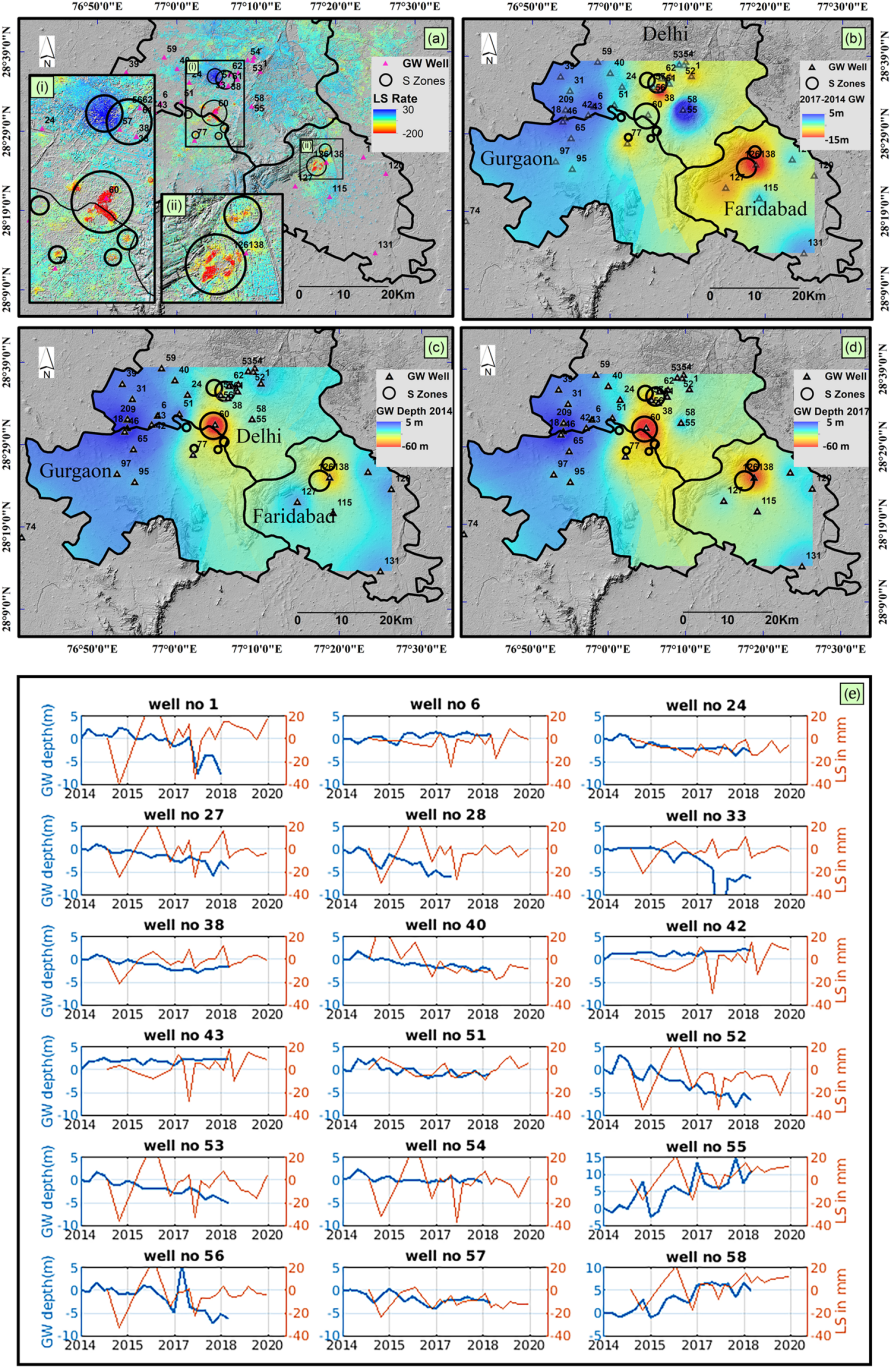
The relation between land subsidence and groundwater extraction. (**a**) represents the deformation velocity in a vertical direction (mm/year) estimated from PS-InSAR. The small subsidence features are zoomed in (**a**-**i**, **ii**). The subsidence zone is demarcated using black circles as shown in (**a-i**, **ii**). (**c**) and (**d**) represents the groundwater depth of 2014 and 2017 respectively. (**b**) represent the change in groundwater level from 2014 to 2017. The demarcated S-Zones are overlaid on the groundwater depth map of 2014 and 2017. (**e**) represents the time series of deformation in mm (orange color) and groundwater-in situ measurements (blue color) for the wells displayed as black triangles in (**a**)–(**d**). (**a**–**d**) were generated in ArcMap software (Version 10.4; copyright and licensed by ESRI https://desktop.arcgis.com/).

The Comparison between InSAR results and in-situ groundwater data indicates a strong correlation between the two. In Fig. 5a, regions undergoing significant deformation/uplift, are demarcated using black circles (S-Zones) and are overlaid on the groundwater depth map of 2014, 2017 and the rise/fall in the groundwater levels in Fig. 5c, d, b respectively. Interestingly, the S-zones exactly overlap the region with deep groundwater levels. The two major deformation features, i.e., Kapashera (subpanel (i) in Fig. 5a) and Sanjay Gandhi Memorial Nagar, Faridabad (subpanel (ii) in Fig. 5a) lies in the regions of deepest groundwater levels across the NCR region. Furthermore, the rate of groundwater depletion in Faridabad is found to be exceptionally high (5 m/year) and serves as reliable evidence to explain the high subsidence rates in Kapashera and Faridabad.

A comparison of subsidence time series and groundwater in-situ data is shown in Fig. 5e. The blue line represents the groundwater depth (in meters), while the red line represents the ground subsidence (in mm). The groundwater in-situ data was available from 1996 to 2018; however, we retrieved the data from 2014 to 2018 to collate it with InSAR observations (refer Fig. M5 in Supplementary). Groundwater behavior, at different locations, differs significantly. For some wells, the groundwater level shows a declining trend, while it is stable or follows a rising trend at other locations. The intercomparison of groundwater and land subsidence shows some discrepancies. For most locations, the subsidence and groundwater follow a similar trend, for instance, well no 6, 24, 51, 55, 57, and 58. However, at other locations, the groundwater trend does not match with land subsidence. For example, in well no. 56, groundwater level shows a declining trend, however the subsidence is not significant. These discrepancies between groundwater and subsidence may be due to (1) aquifer response to groundwater change—the aquifer response to changes in groundwater level is not immediate. Often a delay is observed between groundwater level changes and soil compaction, which is strictly attributed to the geotechnical and geometrical features of the drainage conditions and the hydraulic properties of soil such as permeability and porosity. (2) Seasonal variation of groundwater^10^, and (3) difference in temporal sampling—the InSAR observations are available every 6th day (considering S1A and S1B satellites), whereas, the groundwater data is acquired only four times a year. The inconsistency between groundwater and land subsidence for few wells in Delhi NCR requires further investigation.

### Subsidence evolution towards international airport Delhi

**Figure 6:**
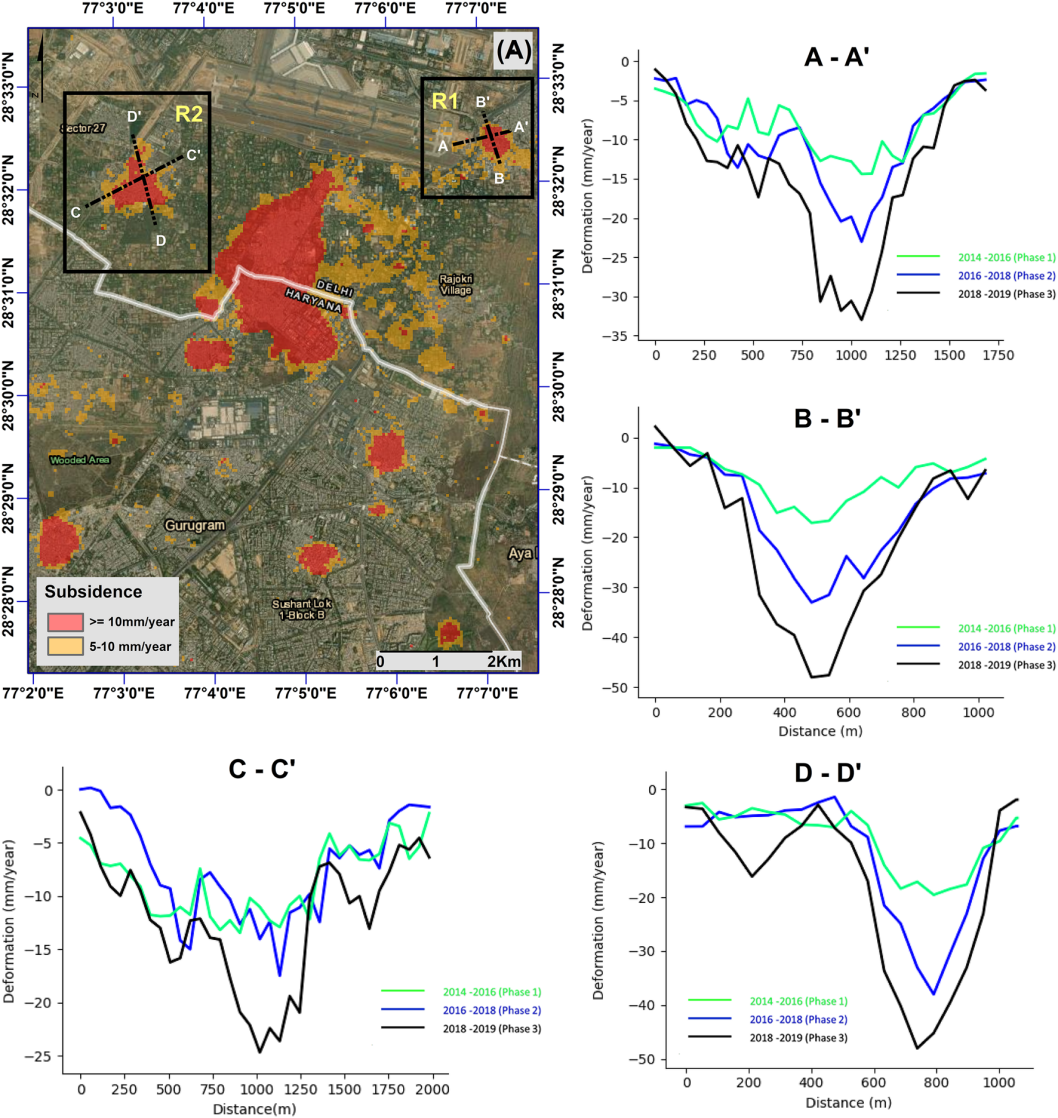
Line profile for the sections A–A′; B–B′; C–C′ and D–D′. The first image shows the area and the subsidence velocity associated with it. The line profile is shown in the figures (A–A′ to D–D′). The green, blue, and black color represents subsidence velocity from phase 1, phase 2, and phase 3 respectively. (′) was generated in ArcMap software (Version 10.4; Base map: World Imagery; copyright and licensed by ESRI https://desktop.arcgis.com/).

The major subsidence feature in the study area, Kapashera is less than 800 m away from the international airport, Delhi (Fig. 6a). Indra Gandhi International (IGI) Airport handles more than 900 flights a day and is the busiest airport in India. In addition, there are a few local subsidence features that are increasing with time. In particular, the Mahipalpur area which is located adjacent to the airport (denoted as R1 in Fig. 6), and Bijwasan Harijan Basti in the south-west direction of the track (denoted as R2 in Fig. 6).

The Mahipalpur area (region R1), is located approximately 500 m from the IGI Airport. The area reports a continuous increase in magnitude and spatial extent of the land subsidence. An east–west section line A–A′ and north–south section line B–B′ are presented in Figure A–A′ and B–B′ respectively. Section line B–B′ shows a significant increase in subsidence velocity over the past five years. The highest rate of deformation was around 15 mm/year in first phase; 30 mm/year in second phase; and approximately 50 mm/year in third phase. Section line A–A′ also reports similar results as that of B–B′. However, a new “V-shape feature” in phase 3 (represented by the black line) suggests a new subsidence funnel near the runway of the airport. It is reported by Ziwen et al.^75^ that such high variability in the subsidence gradient is often unstable, and therefore the two independent subsidence funnels (Fig. 6b, black line) are more likely to join together with time causing damage to the runway and the other infrastructure in Mahipalpur area.

A similar increasing trend of the subsidence velocity is observed in Bijwasan Harijan Basti which is 1.5 km from the runway. Section line C–C′ and D–D′ shows a dominant subsidence funnel rapidly progressing in time. The maximum rate of deformation increased from 15 mm/year in phase 1 to more than 30 mm/year during phase 3 (2018–2019).

The rapid increment in the spatial extent and the magnitude of subsidence poses a very serious threat to the airport. It is, therefore crucial to monitor the subsidence in this area on a regular basis.

### Other consequences of land subsidence in Delhi NCR

Subsidence causes subtle changes in land gradient which can impact storm drainage or sewer lines that flows under gravity^76^. In many parts of Delhi NCR, the underground utilities (sewer lines, water mains, etc.) are laid beneath the roads due to the shortage of space. Whenever there is damage to these utilities, the water seeps in making the lower soil layer soft and after some time, the roads cave in^77^.

Old Delhi-Gurgaon Road, a 7.5 km-long stretch linking Delhi and Gurgaon is one of the busiest roads in the area and is full of cracks and potholes. Times Of India—a news agency reported that even after spending millions of rupees multiple times, the condition of the road never improved^78^. Our InSAR analysis results show that this road has subsided by more than 70 cm in the past five years (Fig. D1 in supplementary). It is built on the top of a 30-year-old underground cement sewer pipeline. The civic authorities can look into the possibility of subsidence-induced gradient change of the sewer pipe resulting in leakage of sewage, breaking open the pavement, and deteriorates the road condition. However, further investigation is required to study the impact of differential subsidence on the utilities. The second major indirect consequence of the subsidence is aggravated flood risk and more frequent rainfall-induced floods waterlogging. Several studies have emphasized the effect of land subsidence on the increased flood extent and inundation depth in Jakarta^79^, Semarang (Indonesia)^80^ and Shanghai (China)^81^. Similar results were observed in Delhi and Faridabad where waterlogging was observed just after minutes of rainfall and flood intensified during the past years. Kumar et al.^82^ recommended several structural and nonstructural measures to mitigate flood risk in Delhi NCR. Concerned authorities and flood planners should also consider local subsidence features during flood modelling, which is currently not incorporated in the present model of flood risk management.

### Hazard, vulnerability, and risk assessment—identifying the risk of ground movement

The above results show an increasing temporal and spatial extent of land subsidence over the past 5 years and likely to increase as residents of NCR heavily depend on groundwater^58^. Certain regions in the study area are at greater risk than others due to the uneven demographics, geology, and anthropogenic activities. Risk—in simple words, is a situation that involves exposure to danger and is formally expressed as the function of Hazard, Vulnerability, and exposition^52^. In the context of the current study, hazard include natural or anthropogenic events that may cause ground instability, whereas vulnerability incorporates the fraction of elements that are likely to get affected due to the occurrence of hazard.

**Figure 7:**
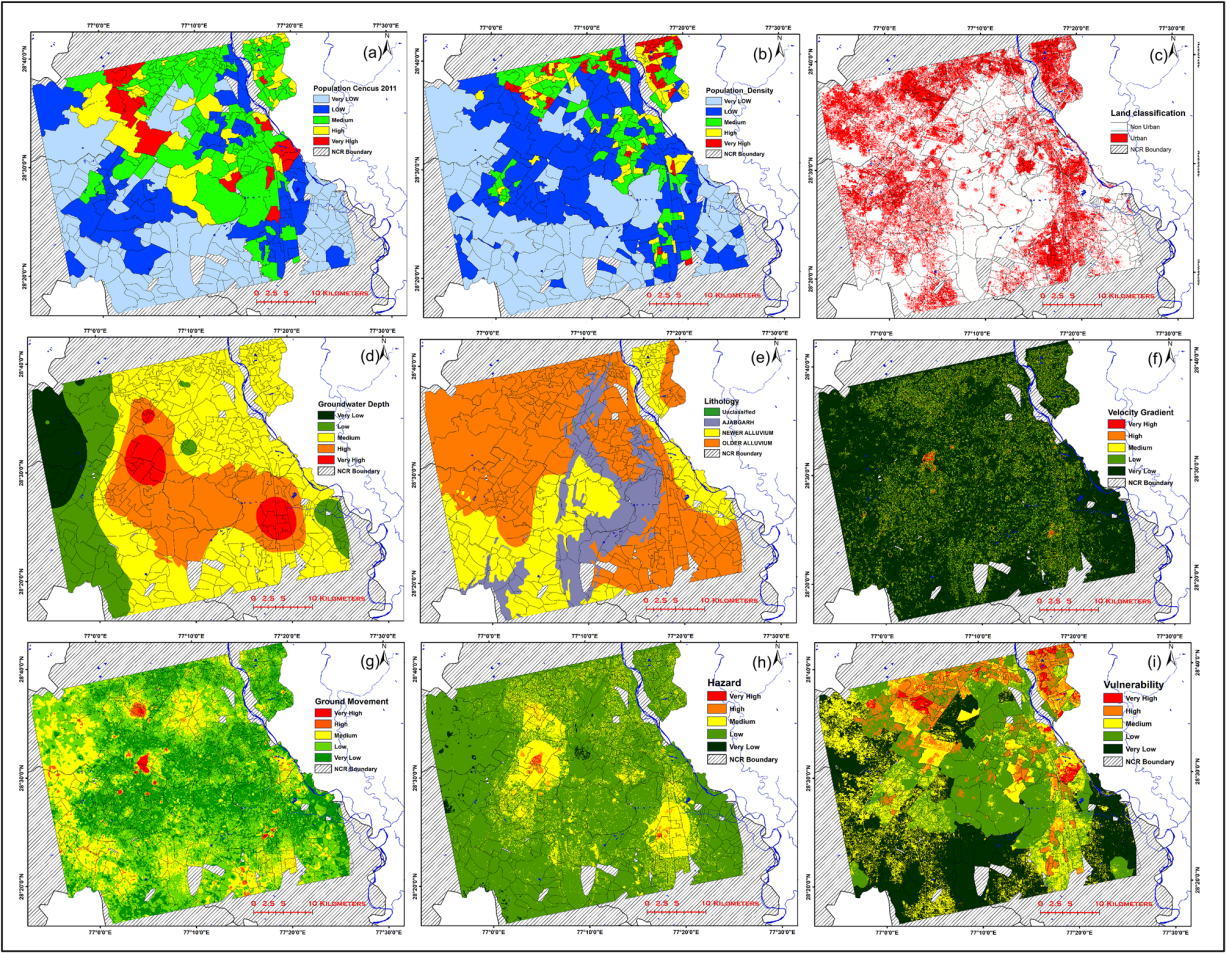
Hazard map, Vulnerability map and Indices used for Hazard Vulnerability and risk analysis. (**a**) Population Census 2011, (**b**) Population Density, (**c**) built up/non built-up areas, (**d**) Groundwater depth; and for vulnerability, (**e**) Lithology, (**f**) velocity gradient, (**g**) ground movement, (**h**) Hazard and (**i**) Vulnerability. The maps are classified into five classes and ranging from very high (red) to very low (green). Maps were generated in ArcMap software (Version 10.4; copyright and licensed by ESRI https://desktop.arcgis.com/).

Figure 7 shows several indices used in the study to generate classified risk map. Hazard map which combines the risk elements represented by groundwater extraction, lithology, subsidence gradient, and subsidence velocity (Fig. 7d–g respectively) is represented in Fig. 7h. The map is classified into 5 classes ranging from very high to very low hazard. As expected, the highest level of hazard is reported in areas of high groundwater extraction particularly from the alluvial aquifers and high subsidence velocity. It also takes into account the effect of subsidence gradient, which is the major factor responsible for cracks in buildings. Vulnerability, on the other hand, is represented by population, population density, and land cover (Fig. 7a–c). Figure 7i shows the classified vulnerability map. The highly vulnerable areas are in the south-east and north-western part of the region and primarily include built-up areas with high population, and population density. Any casualties in these areas may cost a huge loss of life and economy.

**Figure 8:**
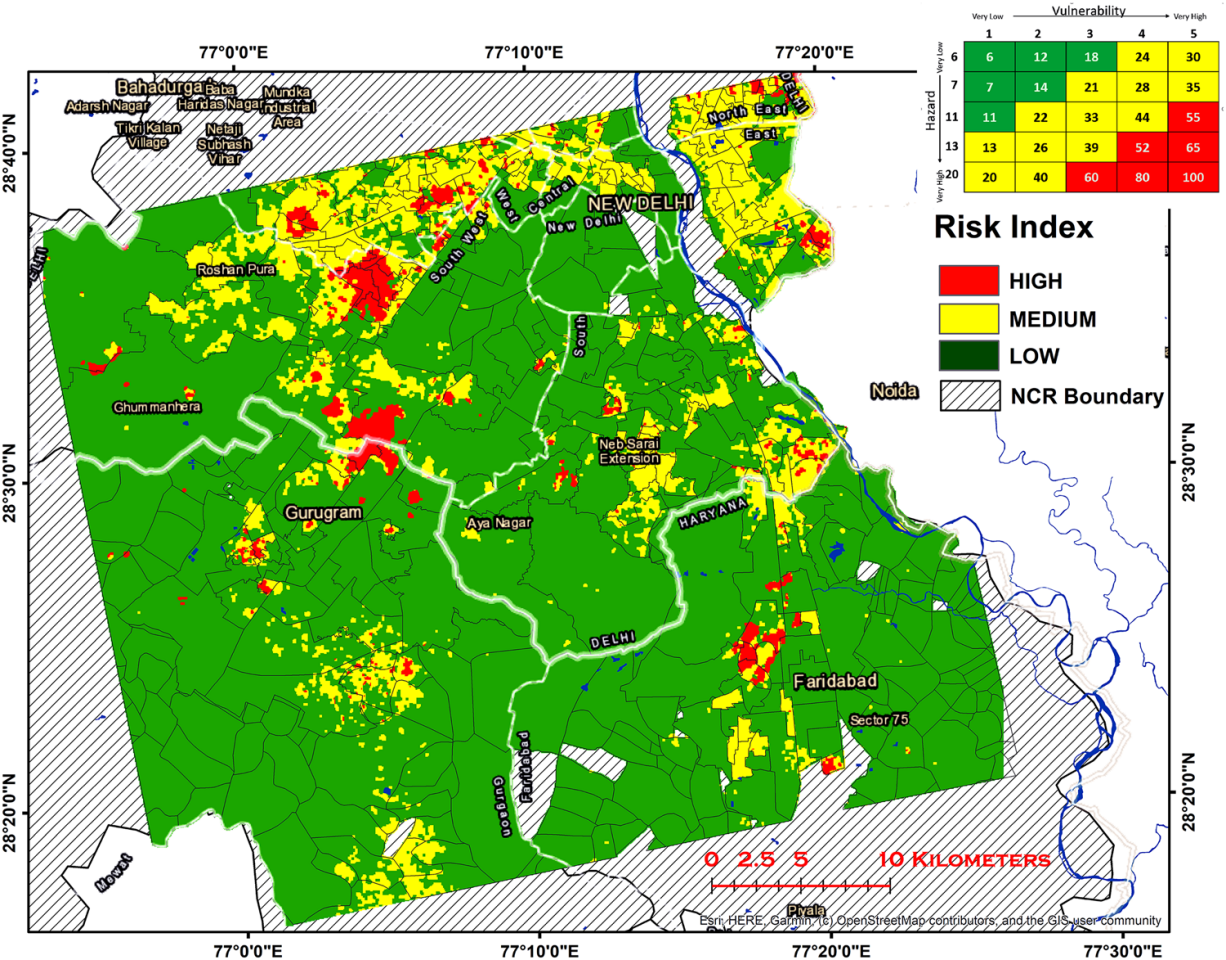
Risk map classified into 3 classes viz, high, medium and low. The matrix in the top right corner represents the risk matrix. Hazard and vulnerability maps (classified into 5 classes) are reclassified and assigned numbers such that the product of any two numbers (one from hazard and other from vulnerability) is unique. Regions with high vulnerability and hazard are assigned high risk and low vulnerable—low hazardous regions are assigned low risk. Almost 100 km^2^ of area is found to be under high risk of ground movement. Figure was generated in ArcMap software (Version 10.4; copyright and licensed by ESRI https://desktop.arcgis.com/).

Both the classified risk and hazard map are then combined using a multiplicative matrix approach. The regions that fall under high vulnerability and high hazard is classified as high risk. Similarly, low hazard and vulnerability areas are identified as low risk. Figure 8 illustrates the map classified into three levels of risk pertaining to ground movement. The regions of the higher risk in NCR are mainly distributed in the northern-west and south-eastern part of the region under investigation. A total area of approximately 100 km^2^ is found to be under high risk of ground displacement. Most of these are urban areas with a high population density and high subsidence gradient. A higher gradient in the urban areas causes higher shear stress and consequently more damage to infrastructure^51^. The areas under high risk that require immediate attention include Bijwasan, Samlkha, Kapashera, Sadh Nagar 1, Bindapur, and Mahavir enclave from Delhi; Dundahera, Sector 22A, and Block C from Gurgaon; and Pocket A, B, C of Sanjay Gandhi Memorial Nagar, from Faridabad. The risk map shown below can be further improved by (1) Considering high-resolution hydrogeology and a detailed aquifer map of the area, (2) High resolution land subsidence estimates, and land cover data to detect large subsidence gradient on building scale. (3) Increasing the spatial and temporal sampling of in-situ groundwater observations.

## Conclusion

This study investigated land subsidence in Delhi National Capital Region using the PSInSAR technique. The land subsidence rate has been quantified through the Sentinel-1 ascending and descending pass SAR datasets acquired between 2014 and 2020. To minimize the effect of temporal decorrelation, the time period has been divided into three phases, each covering a different period. The individual time series from different phases were eventually merged together to form a long-term displacement time series between 2014–2019. A total area of approximately 11 km^2^ was found to undergo continuous deformation since 2014. It included Kapashera (Delhi–Haryana border), Dwarka, and Sanjay Gandhi Memorial Nagar in Faridabad. The maximum rate of subsidence exceeded 17 cm/year in Kapashera, and 7 cm/year in Faridabad during 2018–2019 period. Besides this, there were small subsiding areas, which expanded over time. For instance, Samalkha and Mahipalpur located in the immediate vicinity of IGI Airport Delhi reported an increased rate of subsidence in recent years.

The comparative analysis of groundwater depth between the years 2014 and 2017, reports the highest groundwater depth of 80 m in Kapashera, followed by 60 m in Faridabad. A high groundwater depletion rate was also observed in these areas. Interestingly, these locations exactly match with significant subsidence areas, investigated using time series InSAR. This suggests that groundwater extraction is the primary cause of land subsidence. Considering the population, land use, lithology, groundwater depth, subsidence, and subsidence gradient, the study area was classified into high risk, medium risk, and low risk, using the hazard-vulnerability approach. A total area of approximately 100 km^2^ is found to be at high risk of ground displacement. It includes Bijwasan, Samlkha, Kapashera, Sadh Nagar 1, Bindapur, and Mahavir enclave from Delhi; Dundahera, Sector 22A, and Block C from Gurgaon; and Pocket A, B, C of Sanjay Gandhi Memorial Nagar, from Faridabad. Most of these locations have a high population density and do not access to the pipe water supply. Hence, the illegal extraction of groundwater is very common in these areas.

In general, the groundwater scenario of Delhi -NCR is really challenging. There is a huge gap of 750 million liters a day between the demand and the supply, which leads to the extraction of the water from the underground^33^. Rainwater harvesting is one solution to the problem^83^. Delhi receives an average annual rainfall of 611 mm, mostly in July, August, and September. Harvesting rainwater will not only bridge the gap between demand and supply but will also replenish the falling groundwater levels. The groundwater recharge can reduce the increasing rate of land subsidence and can mitigate the risk associated with subsidence.

## Datasets and methodology

In this study, we utilized the full temporal extent of the Sentinel-1 mission (S1A from 2014–2020; S1B from 2016–2020) acquired in IW mode. The region of interest lies on track 136 and track 27 for descending and ascending pass respectively. A total of 219 differential interferograms from 225 Sentinel-1 SLC images were generated in ascending and descending directions to monitor the subsidence in Delhi-NCR. Refer to Table AT1 and AT4 in the appendix for the complete list of Sentinel dataset used in this study. Space Shuttle Radar Topography Mission (SRTM) Digital Elevation Model (DEM) of 90 m resolution was downloaded from USGS Earth Explorer. The Geology and lithology data was acquired from the Central Ground Water Board Reports on Aquifer Mapping and Management Plan^84,85^ and Geological Survey of India (GSI). The data is available in the public domain and can be accessed from the Bhukosh portal of GSI (http://bhukosh.gsi.gov.in/Bhukosh/Public). The data of groundwater levels was acquired from Central Ground Water Board (CGWB), India and is taken four times a year—January, April/May, August, and November; this data can be accessed from the Water Resource Information System (WRIS) portal https://indiawris.gov.in/wris/. The population data for Delhi, Faridabad, and Gurgaon were downloaded from the primary census abstract of 2011 (Census PCA Delhi, 2011). https://censusindia.gov.in/pca/pcadata/Houselisting-housing-Delhi.html. Since the digital census section maps of the areas are not available, we created the shape-file using the digitization technique using ArcGIS. It is discussed in more detail in the supplementary dataset.

The methodology used in this study is explained below:

### Splitting the time period

Here we attempted to monitor land subsidence from 2014 to 2020 using Sentinel-1 ascending and descending pass datasets. Temporal decorrelation or loss of coherence with time is a major limitation in InSAR^86^. Therefore, we divided the time period into three phases such that the maximum temporal baseline between the master and slave image is limited to 1.2 years. Refer to Fig. M1 in the supplementary to visualize the effect of temporal decorrelation and Fig. M2 for the spatiotemporal changes for the coherence histogram and the cross comparison of the mean coherence values of the three phases in ascending direction. Figure M3 in supplementary shows the mean coherence plots for the three phases.

Table 1 shows the datasets used for the three phases in ascending and descending pass and the respective master image.

**Table 1:** The duration of six phases for ascending and descending and their respective master image.

Orbital direction	Phase	Dataset	Master Image
Descending	Phase 1	Oct 2014–Dec 2016	Feb 2016
	Phase 2	Aug 2016–Sep 2018	Sep 2017
	Phase 3	Aug 2018–Jan 2020	Aug 2019
Ascending	Phase 1	Nov 2014–Dec 2016	Dec 2015
	Phase 2	Oct 2016–Oct 2018	Aug 2017
	Phase 3	Aug 2018–Jan 2020	Jan 2019

### Interferogram generation

Sentinel-1 Single Look Complex (SLC) datasets were downloaded from the Alaska SAR facility to perform Interferometry. The processing for the generation of interferogram was carried out using the Sentinel Application Platform (SNAP) toolbox. The information about the satellite’s precise position during the data acquisition was provided by orbit auxiliary data was used to remove the flat-earth phase component. The orbital position of the satellite, if inaccurate can cause a phase-ramp in the interferogram.

The stack of the SLC images was co-registered w.r.t one master image. Selecting a master image is a crucial step in Interferometry, particularly in PS-InSAR. It should be selected in such a way that the variation of the perpendicular and the temporal baseline is minimum and should also consider the effect of Doppler central frequency^87,88^. The final step in this selection is to check whether the optimal master is affected by the atmosphere. This is done by generating a few interferograms with the same master image. If a similar atmospheric pattern appears in all the interferograms then the current master image is removed from the stack, and a new master is estimated based on the temporal and perpendicular baseline. This process is repeated until an atmosphere-free master image (by visual analysis) is found. Figure M4 in supplementary represents the time vs baseline plot for the six stacks. Each circle and line represent the acquisition date and the interferogram respectively. Owing to the different viewing geometry and sensor height, the same target on the ground may appear in the different pixels in the master and slave image. To overcome this discrepancy, slave images are adjusted in a way that they are aligned with the master image to an accuracy of 0.001 of pixel size in the azimuth direction^89^. The SAR images were then stacked together with the master image on the top of the stack. To further improve the accuracy of co-registration, we applied an Enhanced Spectral Diversity (ESD) operator, which corrects range and azimuth shifts in the slave image. It is recommended to check the results after co-registration using RGB representation (TOPS Interferometry Tutorial). After co-registration, debursting was done to merge the separate bursts seamlessly into a single image following by Interferogram generation. Phase change due to topography was calculated using 90 m SRTM DEM to simulate an interferogram and subtract it from the interferometric phase.

As the flat earth phase and topographic phase were removed, the interferogram now only contains the phase due to deformation, atmosphere, and noise. Atmospheric phase delay is due to significant changes in temperature, pressure, and humidity between the two SAR acquisition which changes the refractive index of the atmosphere. It was further estimated and removed using the time series of Interferogram.

### Persistent scatterer analysis

Persistent Scatterer Interferometry or PSI was first developed by Ferretti in 2000^67,90^. It assumes that every interferogram contains a few coherent pixels whose phase can be exploited for deformation analysis. These points are known as ‘Persistent Scatters’ or PS points. The identification of these pixels is based on ‘amplitude dispersion Index (ADI)’ which is the ratio of the standard deviation to the mean of the amplitude (Eq. 1)^90^.

1 $$D_{A} = \frac{{\sigma_{A} }}{{\mu_{A} }}$$

here $\sigma_{A}$ and $\mu_{A}$ are the mean and standard deviation of amplitude. All the pixels having $D_{A}$ less than 0.4 was selected as potential PS points^67^. A total of 4,098,386 PS points has been selected during the processing of the phase 3 descending dataset. The phase noise of the PS points was estimated by removing the spatially correlated phase and spatially uncorrelated DEM error. The points with low phase noise were considered as final PS candidates, whereas the points with high noise were dropped from further analysis. In the end, 2,628,264 PS points were selected for further processing. An important point to note here is that all this processing was done without multi- looking and hence at the highest resolution possible. It maximizes the chance of identifying stable pixels amongst noisy pixels.

### Phase unwrapping, time series and velocity estimation

After the selection of PS points, phase unwrapping is performed using the 3D unwrapping method^88^. However, before unwrapping, the phase of the PS points is filtered in the spatial domain to suppress the effect of spatially uncorrelated terms (Gaussian noise). As filtering is done using a Goldstein filter in continuous phase space, it requires the resampling of the irregular placed PS points over a regular spaced grid^91^. The grid size is controlled using unwrap_grid_size in STaMPS. This is a crucial parameter and must be selected in a way that balances the real subsidence estimation and computing capacity. We tested two different unwrapping grid size i.e., 200 m and 50 m over an area with high deformation gradient. It can be observed that for the case of grid size of 200 m, the time series of deformation shows many jumps and the maximum deformation was only 3 cm/year in the line-of-sight direction. On the contrary, for the grid size of 50 m, we see a near-linear time series of displacement with a maximum deformation of 14 cm/year. The 200 m grid size highly underestimates the deformation due to phase unwrapping errors. (Refer Fig. M6 in supplementary materials). Upon analyzing the wrapped interferograms, we observed a constant change in the no. of fringes (fringe count decreases until 28.08.2017 (master image) and then increases) representing a continuous deformation as with the case of grid size 50 m. This verifies our hypothesis that a grid size of 200 m underestimates the deformation signal due to the phase unwrapping error. Eventually, the grid size of 50 m is selected for further InSAR processing and time series analysis.

Then we estimated the spatially correlated look angle error (SCLA), orbit error, and master atmosphere. The SCLA error majorly accounted for errors in DEM, and incorrect mapping of the DEM into radar-coordinates^92^. Furthermore, we estimated phase-based linear tropospheric correction (aps_linear) using Toolbox for Reducing Atmospheric InSAR Noise (TRAIN)^93^. After removing all the uncertainties associated with InSAR (DEM error, atmospheric, orbital error, etc.), the phase values were converted into displacement using Eq. (2)

2 $$h = \frac{\lambda }{4\Pi } \emptyset$$

Here, ‘h’ is the displacement in line of sight (LOS) direction; and $\emptyset$ is the unwrapped phase and $\lambda$ wavelength. Errors that are neither correlated in space nor in time were treated as noise.

The displacement measured using the InSAR technique depends on the imaging geometry and is described as a line of sight (LOS) displacement. The LOS displacement can be decomposed into horizontal (east–west) and vertical (up-down) using Eq.(3) by considering both descending and ascending InSAR datasets^43^.

3 $$\left( {\begin{array}{*{20}c} {d_{asc} } \\ {d_{des} } \\ \end{array} } \right) = \left( {\begin{array}{*{20}c} {\cos \theta_{asc} } \\ {\cos \theta_{des} } \\ \end{array} \begin{array}{*{20}c} { - \cos \alpha_{asc} } \\ { - \cos \alpha_{des} } \\ \end{array} \begin{array}{*{20}c} {\sin \theta_{asc} } \\ {\sin \theta_{des} } \\ \end{array} } \right) \left( {\begin{array}{*{20}c} {d_{ver} } \\ {d_{hor} } \\ \end{array} } \right)$$

Ignoring horizontal component, we can derive vertical velocity from LOS using Eq. (4)

4 $$d_{ver} = \frac{{d_{LOS} }}{{cos_{\theta } }}$$

here $\theta$ is the incidence angle; $d_{ver} ,\;d_{LOS}$ are the displacement in vertical and line of sight directions respectively. In our case we have used the line of site deformation from ascending dataset to calculate the vertical deformation.

### Merging time-series

The time span of the Sentinel-1 dataset was divided into three phases, with some overlap in between (Fig. M1 in supplementary). To obtain the merged time series of a particular point, we first generated the individual time series of that point from the three phases. Then the offset between the displacement values for the overlapping period was calculated. This offset was then added to all the displacement values of the succeeding phase resulting in a continuous time series from 2014 to 2020. The rate of subsidence was further calculated by fitting an appropriate model to the time series dataset.

### Groundwater analysis

To investigate the relationship between groundwater extraction and land subsidence, we analyzed the in-situ groundwater data along with the InSAR-derived subsidence results. The in-situ groundwater data provided by the Central groundwater board is first preprocessed and converted into GIS readable format. The groundwater data were available from 1996 to 2018; however, we retrieved the data from 2014 to 2018 to collate it with InSAR observations. There are a total of 139 wells available in the Delhi NCR region out of which only 35 wells have been used in this study due to the poor temporal sampling (more than 70% of the data was missing) of the remaining 98 wells. (Refer to Fig. M5 in the supplementary for more details). The groundwater data, even for the selected 35 wells, was not continuous and had many gaps which were removed by temporal interpolation. Finally, the groundwater data were spatially interpolated using Inverse Distance Weighting (IDW) technique to produce a groundwater depth map for the pre-monsoon 2014 and 2017. This groundwater information was then further analyzed with the InSAR derived subsidence estimates.

### Hazard, vulnerability and risk analysis

Upon reviewing the literature recommendations, and considering the data available in the region, a total of seven comprehensive and representative indices are selected. These include Population (PL), population density (PD), Land Cover (LC), Groundwater extraction (GW), Lithology (LL), Land Subsidence (LS), and Horizontal Subsidence gradient (SG). The PL, GW, and LL are obtained from Census data, Central Groundwater Board CGWB, India, and Bhuvan respectively (Refer data section for more details). PD is calculated by the ratio of population to the geographical area of that particular census section and is expressed in the units of population per unit square kilometer. For the land cover, a stack of 13-sentinel2 bands along with NDVI, MNDWI, and NDBI is used to classify the study area into urban and non-urban classes using maximum likelihood classification technique. Land Subsidence is estimated using sentinel-1 time series InSAR; and horizontal subsidence gradient, which is the change in subsidence velocity (in mm) per unit length (m) is calculated using the slope tool in ArcGIS. These seven indices are georeferenced and are divided into a million grids of size 50 m × 50 m.

In the context of the present work, the risk is defined as a probability of a location suffering from ground instability and the vulnerability of the population from those areas. In this regard, Hazard-Vulnerability, and Risk assessment (HVRA) technique is used to generate a classified risk map. Hazard indicators include LS, HS, GW, LL while vulnerability includes PL, PD, and LC. Weighted overlay analysis is then performed on hazard indices and vulnerability indices separately. Since, the different factors contribute differently to the evaluation process, the weights of each index are chosen differently (Refer Table AT2 and AT3 in supplementary for the weights used in this analysis). The generated Hazard and vulnerability maps are further classified into 5 zones ranging from 0 to 5 (where 0 is very low and 5 is very high). The final risk map is prepared using the multiplicative matrix approach and is classified into three classes viz high, medium, and low risk. Post-processing is done to smoothen the edges and to remove isolated pixels by replacing them with the majority value within their immediate neighborhood.

## Supplementary Information

Supplementary Information.
