# Supplementary material for: Tracking hidden crisis in India’s capital from space: implications of unsustainable groundwater use
Source: Sci Rep. 2022 Jan 13;12:651. doi: 10.1038/s41598-021-04193-9 (PMC8758763; doi:10.1038/s41598-021-04193-9)
Supplement: Supplementary file 1 — Supplementary Information. [file 41598_2021_4193_MOESM1_ESM.docx]

**Supplementary Information**

**
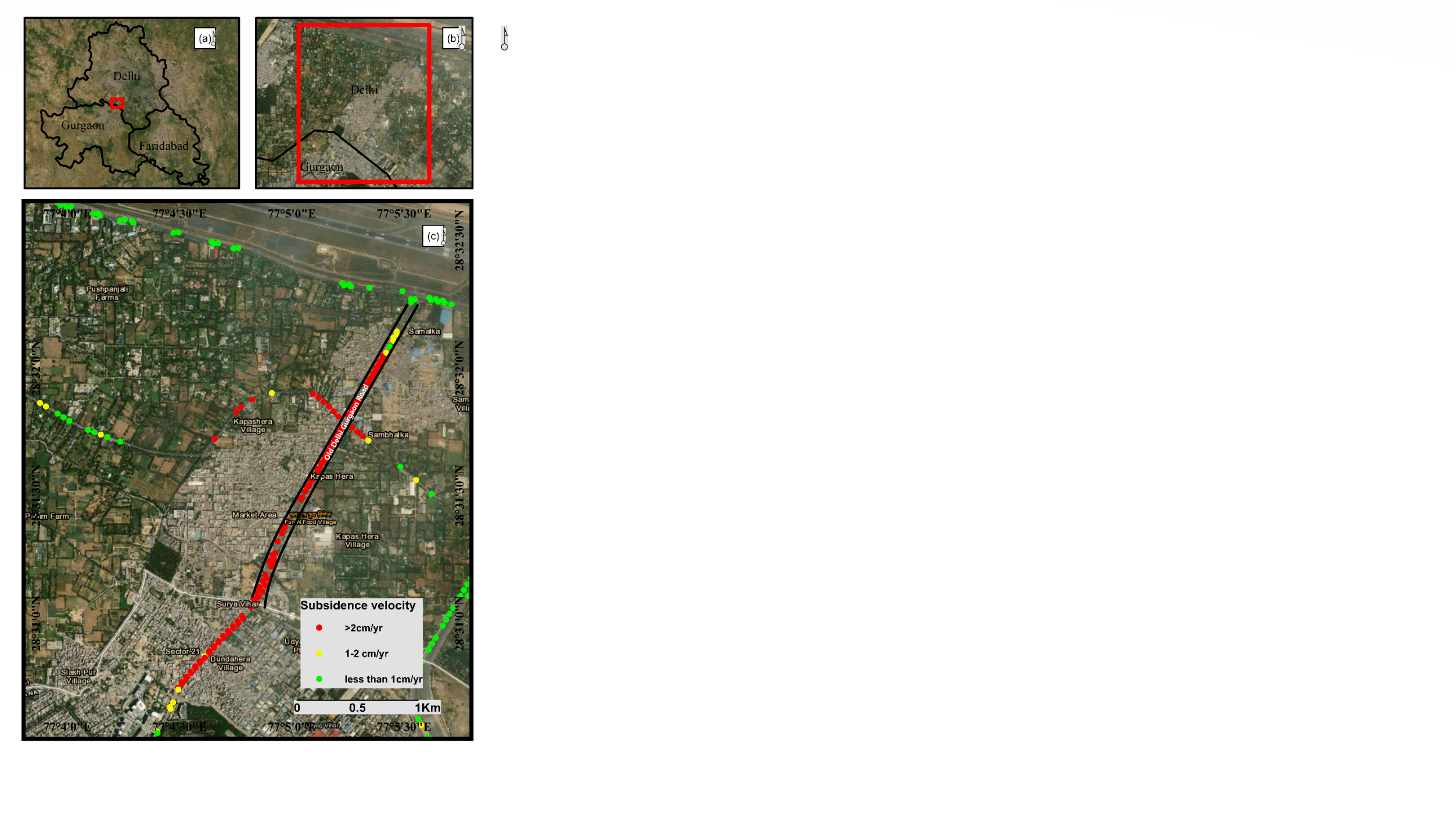
**

**Figure D1** : Subsidence of Old-Delhi Gurgaon road (figure D1-c). The road is one of the oldest stretches and connects Delhi to Gurgaon. InSAR analysis reveals that the road is subsiding by more than 2 cm/year. Figure was generated in ArcMap software (Version 10.4; Base map :World Imagery; copyright and licensed by ESRI https://desktop.arcgis.com/)


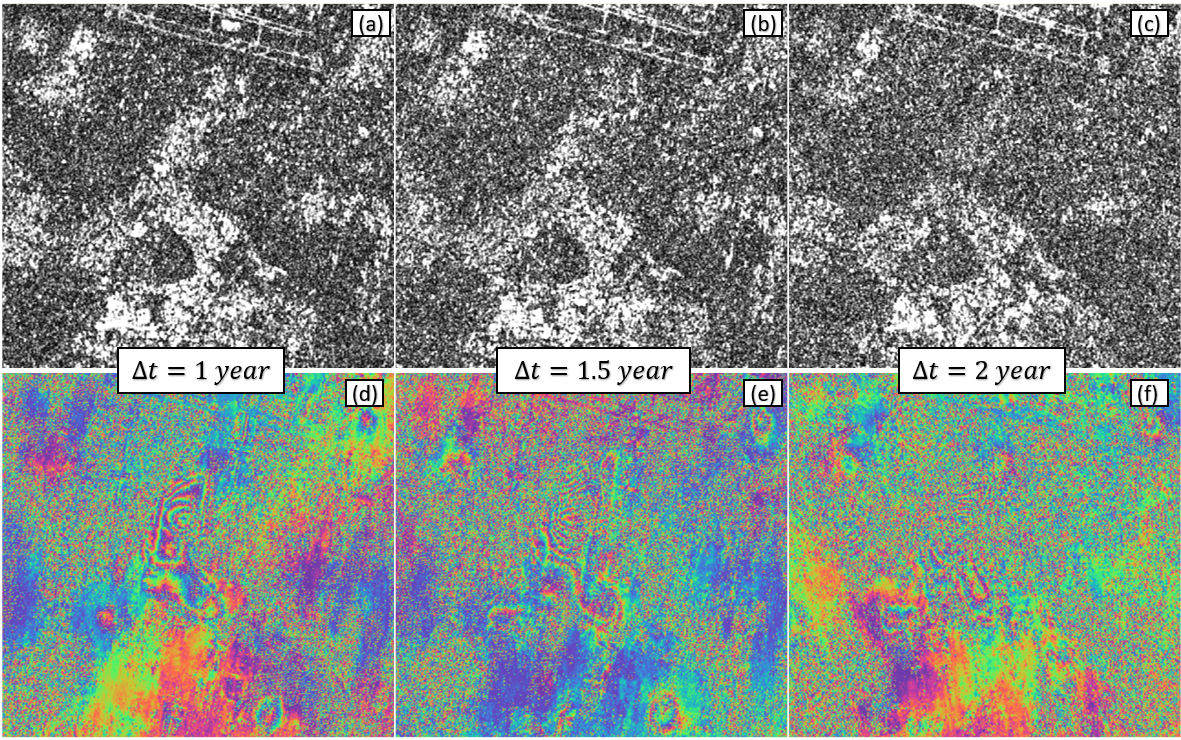


**Figure M1** : Effect of temporal decorrelation: The top panel (a,b,c) represents coherence, and the bottom panel (d,e,f) represents phase having a temporal baseline of 1 year, 1.5 years, and 2 years respectively. Temporal decorrelation (reduction in quality of fringes with time) is clearly visible in c and f. In Figure 1(f) the fringe density becomes too high to be counted and may be misinterpreted as noise, which can lead to the underestimation of the deformation. Therefore, we divided the time period into three phases such that the maximum temporal baseline between the master and slave image is limited to 1.2 years

| **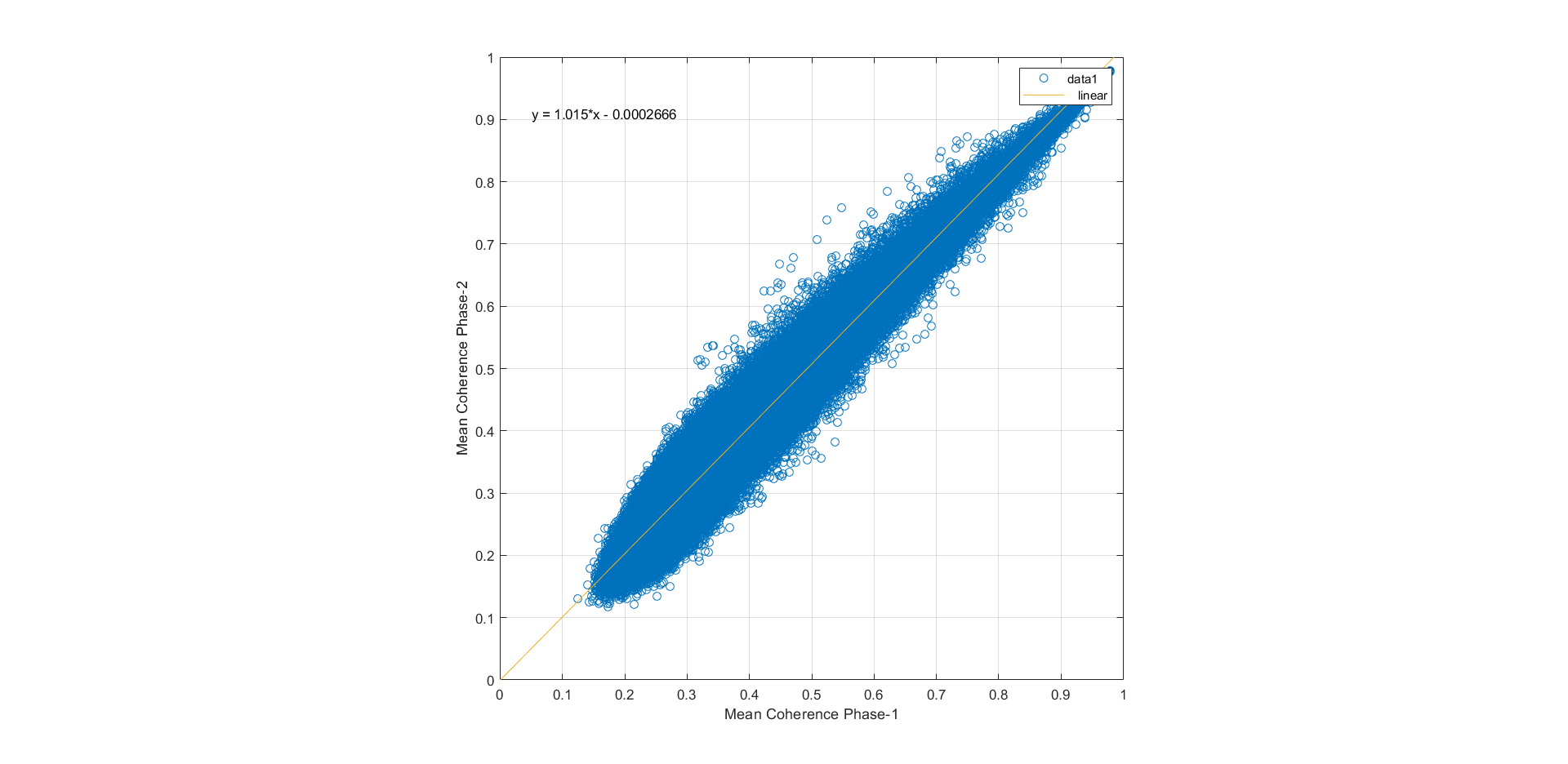**  (a) | **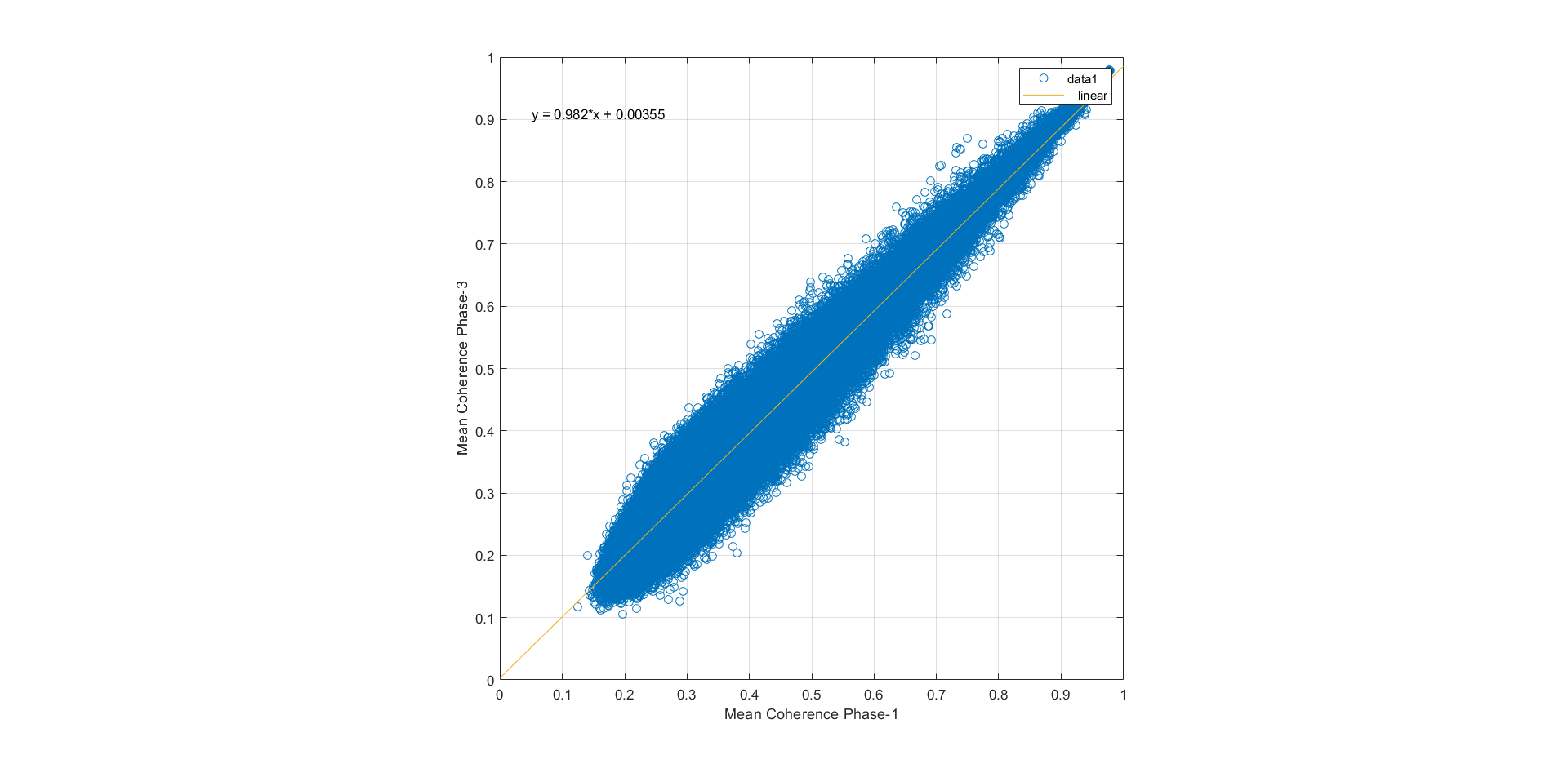**  (b) |
| --- | --- |
| **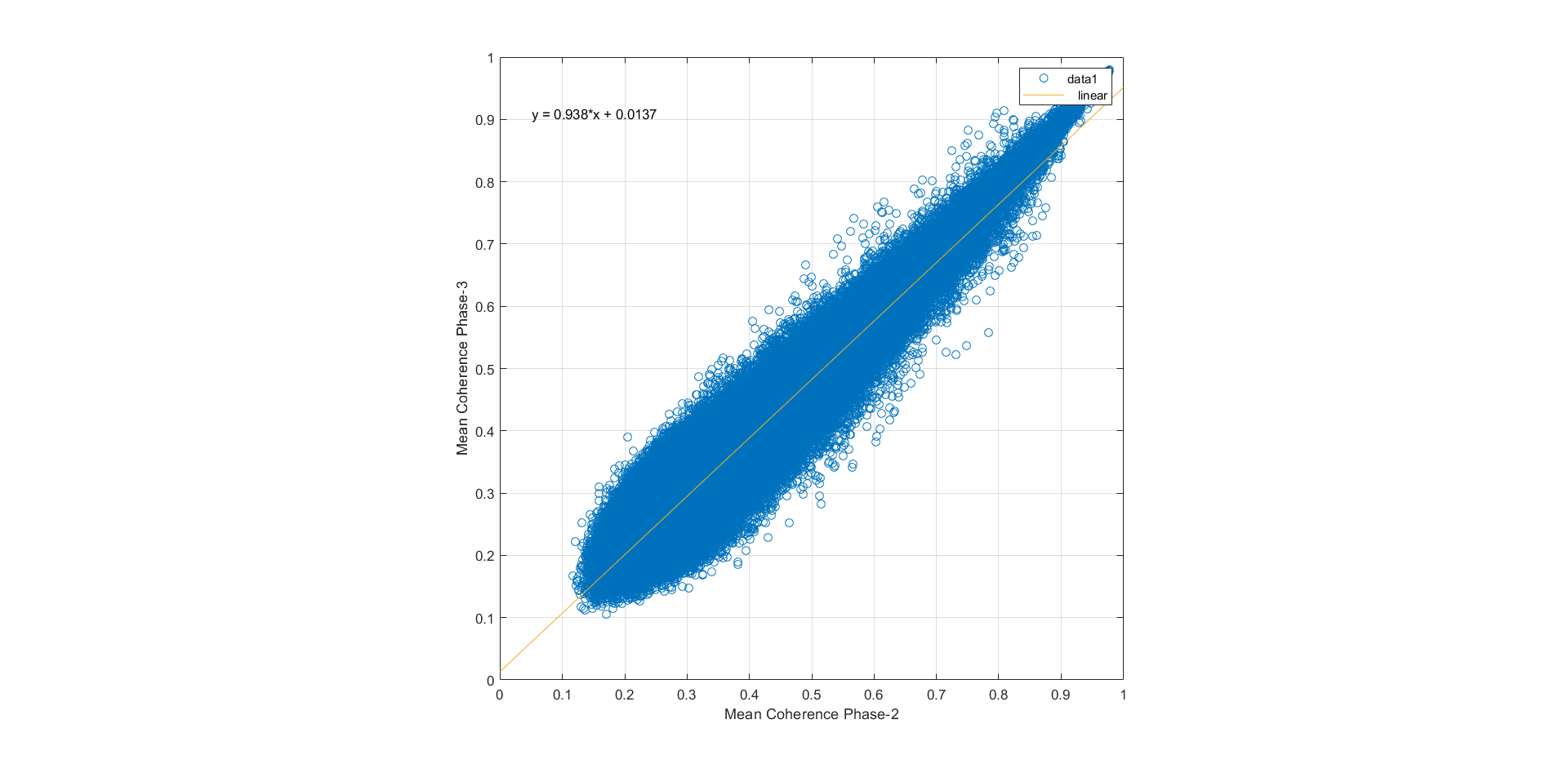** | (c)  (d)  **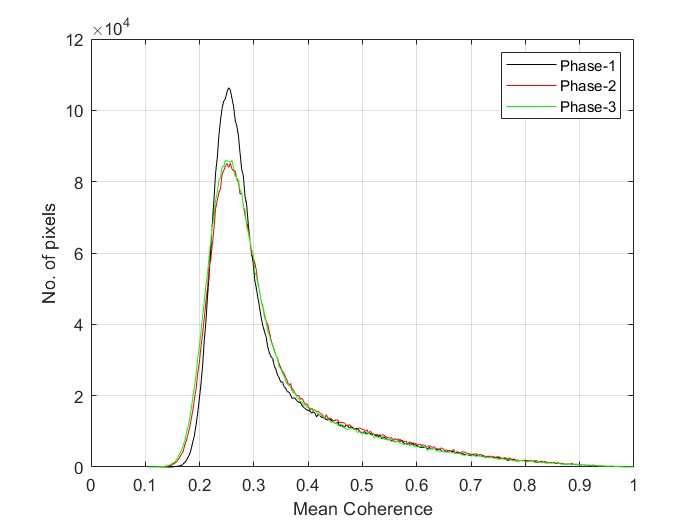** |
|  |  |
|  |  |

**Figure M2**. Cross comparison of mean coherence values of phase-1 (2014-2016), phase-2(2016-2018), and phase3(2018-2020) are shown in subplots a-c. and figure (d) represents the spatiotemporal changes of the coherence histograms of these three phases.

| **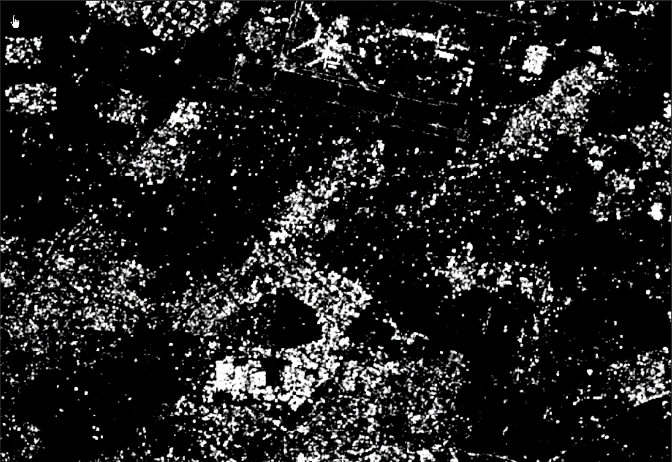**  **(a**) | **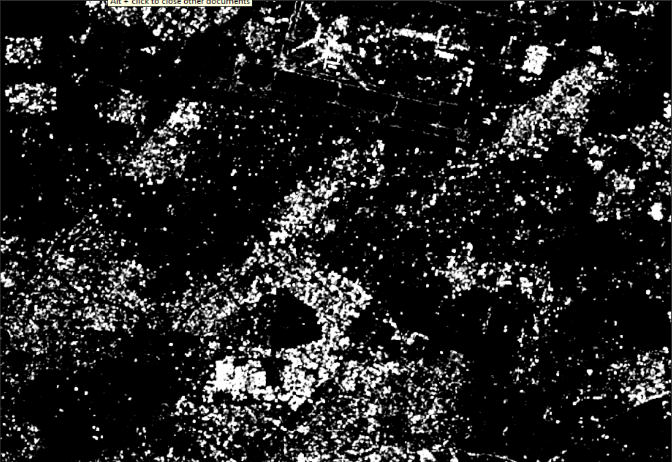**  **(b**) |
| --- | --- |
| **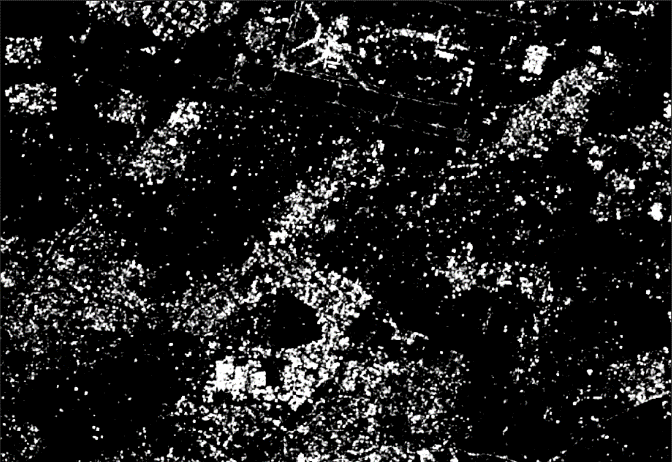**  **(c**) | **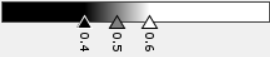** |

**Figure M3**. Mean Coherence values of phase-1 (2014-2016), phase-2 (2014-2016) and phase-3 (2014-2016) are represented in figure (a), (b), and (c) respectively. The color bar is shown in the end panel.


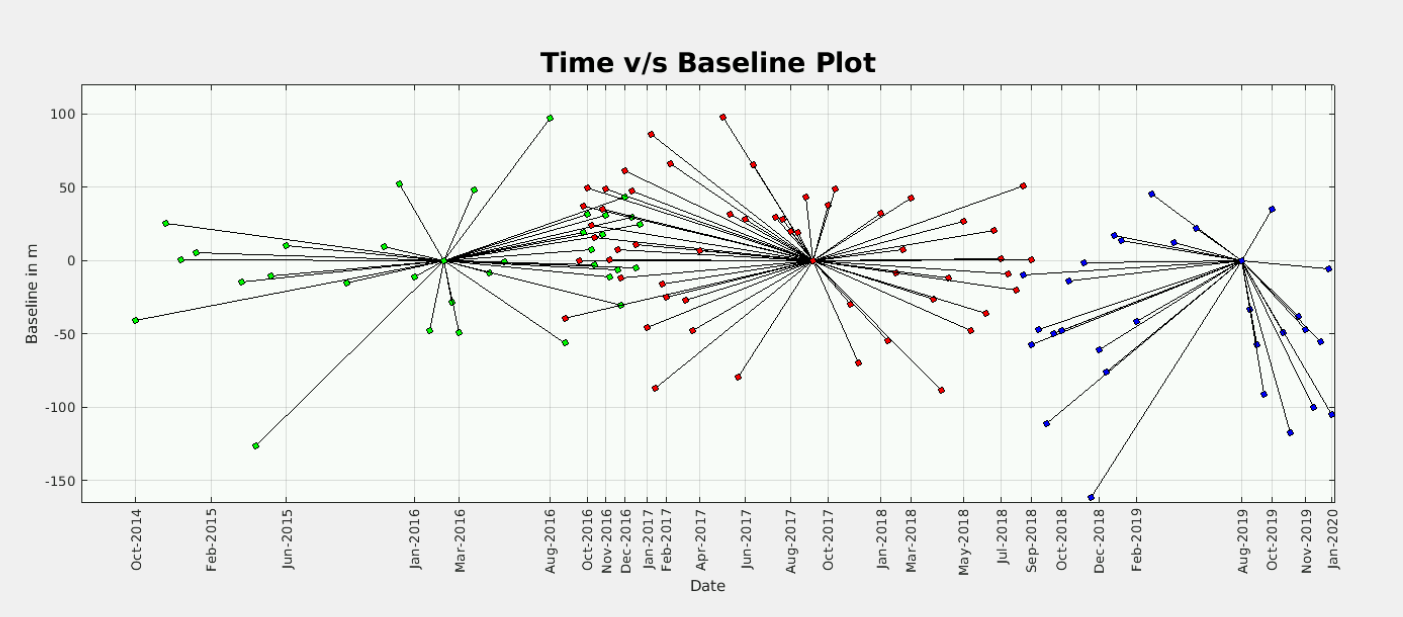

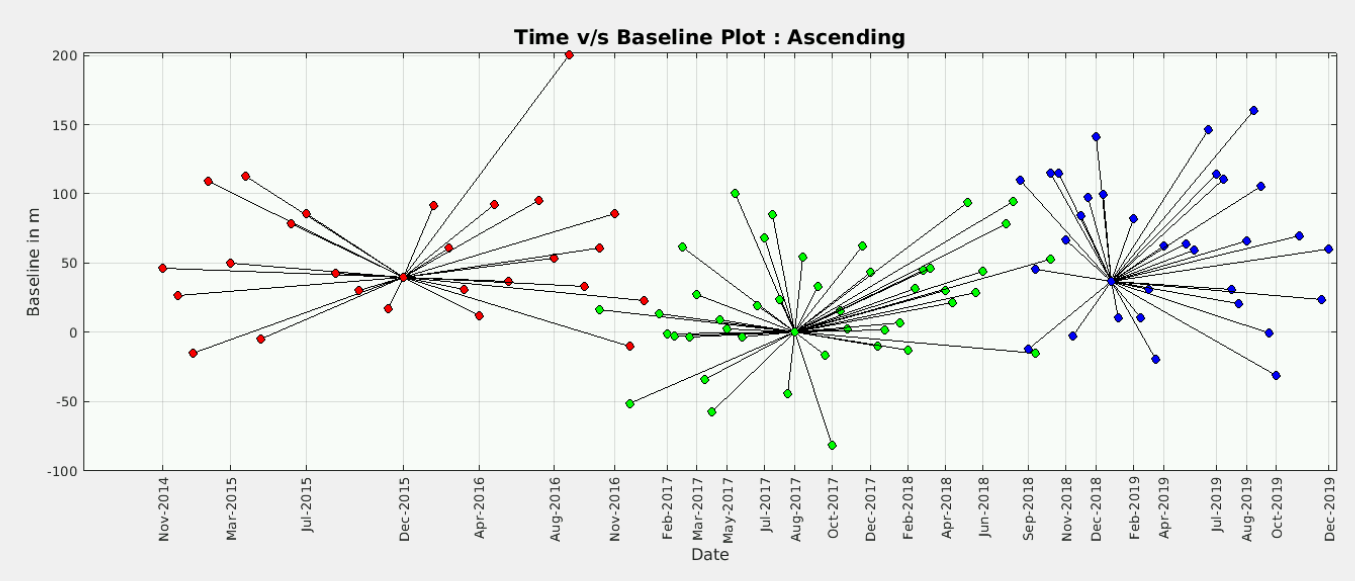


**Fig M4**. Time vs baseline plot of the six different stacks. The top and bottom panels represent the dataset acquired in descending and ascending directions respectively. Each circle and line represent the date of acquisition and interferograms respectively.

**Groundwater insitu data**

Out of 139 wells available in the Delhi NCR region, only 35 wells have been used in this study due to the poor temporal sampling (more than 70% nan values) of the remaining 98 wells. Well numbers 45, 74, 96, 97, 120, 131 have good temporal sampling but can’t be used as it is not located inside the SAR scene. Figure 5. 8 shows the wells available versus wells used, and the area analyzed for subsidence. The groundwater data, even for the selected 35 wells, was not continuous and had many gaps that were removed by temporal interpolation. Finally, the groundwater data were spatially interpolated using Inverse Distance Weightage (IDW) technique to produce a groundwater depth map for the pre-monsoon 2014 and 2017, which were further compared with the subsidence velocity in that region. The results are presented in the results section

**Figure M5** Location of CGWB wells. The yellow circle represents the wells available, and the red circle represents well data used in this study. A lot of wells are not considered due to poor temporal sampling.

**Effect of unwrapping grid size**


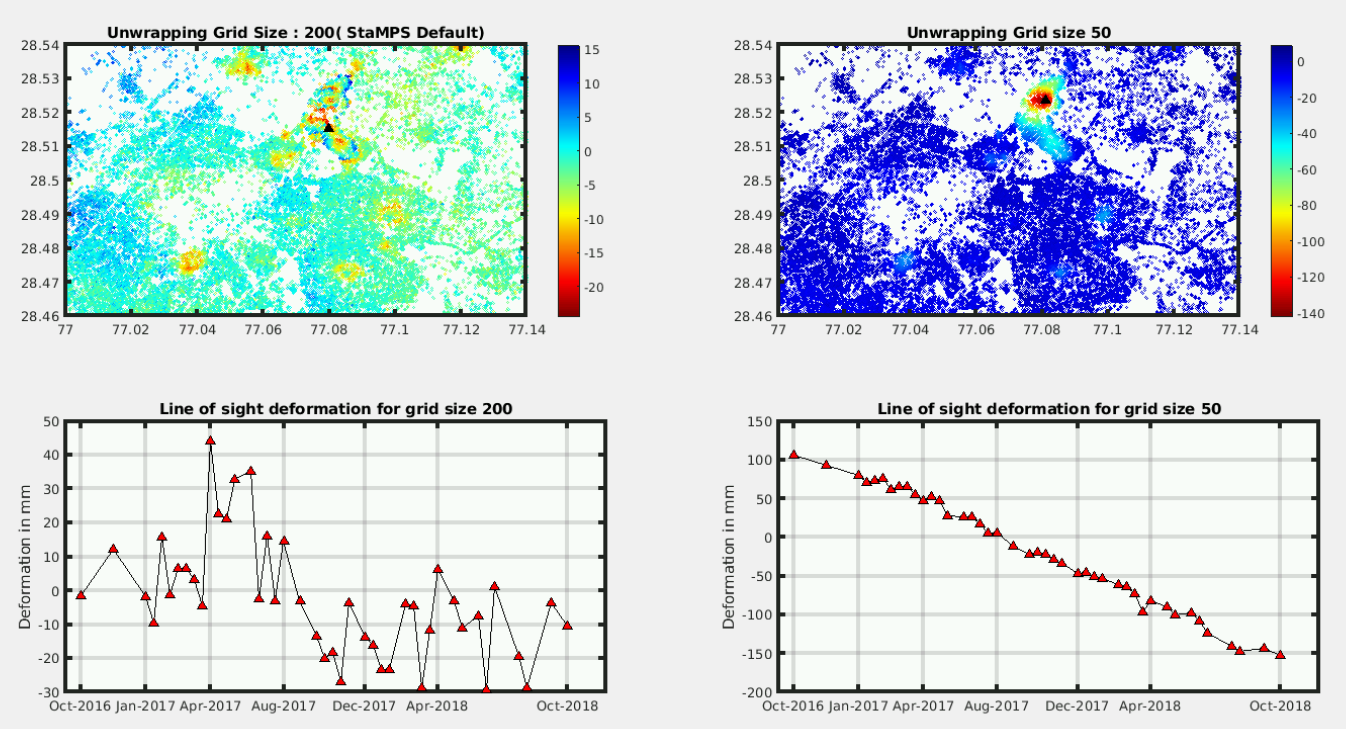


**Figure M6**. Effect of unwrapping grid size; The two images in the first row are obtained after unwrapping using a grid size of 200 and 50 respectively, and their time series is plotted in the next row. Phase unwrapping errors are clearly visible in the first case, i.e. in a grid size of 200.

**Table AT1.** Sentinel-1 data used for processing, Sentinel-1A (S1A) and Sentinel-1B(S1B) orbit pass, first last and the number of images used for the analysis.

| Satellite | Orbit pass | First image | Last Image | No. of Images | Path | Frame |
| --- | --- | --- | --- | --- | --- | --- |
| S1A | Ascending | 24-Nov-2014 | 28-Dec-2019 | 104 | 27 | 86 |
| S1A | Descending | 15-Oct-2014 | 05-Jan-2020 | 111 | 136 | 496 |
| S1B | Descending | 03-Nov-2016 | 30-Dec-2019 | 10 | 136 | 496 |

**Population Dataset:**

The study area includes parts of Delhi, Gurugram and Faridabad and therefore population of the three regions are analyzed separately. In this study, we have used Census 2011 which is the latest available. Digital census section maps of Gurgaon and Faridabad are not are not available in the public domain. Hence, we created the shapefile of the census section by referring the ward maps provided by the Department of town and country planning Haryana. https://tcpharyana.gov.in/CIM/Doc/CMP%20Faridabad%20(Final%20Report).pdf These maps show the census section in Faridabad but do not have any geo-coordinates. Using ESRI map as baselayer in ArcGIS, the ward boundaries provided in the online map were identified and digitized on the base layer as shapefiles and are further reprojected to match the coordinate system of other layers. Affine transformation method used to overcome the observed geometric distortion in the shape file. For Gurgaon, the census boundaries of 2001 were obtained from NASA- Socioeconomic data and application center (SEDAC). The shapefile was then modified to take into account the jurisdictional changes, and the discrepancy amongst census location codes of 2001 and 2011. Delhi wards boundary was downloaded directly in the form of shapefile from Delhi Development Authority (DDA). http://www.dda.org.in/. The population data for Delhi, Faridabad and Gurgaon were downloaded from primary census abstract of 2011. (Census PCA Delhi,2011 ) https://censusindia.gov.in/pca/pcadata/Houselisting-housing-Delhi.html

Table AT2 Weights assigned to the indices used for the generation of hazard map

| Indices | Classes | Weight (%) | Rate (Max 5) |
| --- | --- | --- | --- |
| Ground movement |  | 20 |  |
|  | >= 50 mm |  | 5 |
|  | 30 – 50 mm |  | 4 |
|  | 15 – 30 mm |  | 3 |
|  | 5 – 15 mm |  | 2 |
|  | -5– 5 mm |  | 1 |
| Subsidence Gradient |  | 30 |  |
|  | >= 0.01 % |  | 5 |
|  | 0.009-0.01 % |  | 4 |
|  | 0.006 -0.009 % |  | 3 |
|  | 0.003-0.006 % |  | 2 |
|  | <=0.003% |  | 1 |
| Groundwater depth |  | 30 |  |
|  | >=45m |  | 5 |
|  | 35- 45 m |  | 4 |
|  | 25 – 35 m |  | 3 |
|  | 15 – 25m |  | 2 |
|  | 3 – 15 m |  | 1 |
| Lithology |  | 20 |  |
|  | Older Alluvial |  | 4 |
|  | Younger Alluvial |  | 3 |
|  | Ajabgarh |  | 2 |

**Table AT3.** Weights assigned to the indices used for the generation of Vulnerability map

| Indices | Classes | Weight (%) | Rate (Max 5) |
| --- | --- | --- | --- |
| Population |  | 25 |  |
|  | >= 80000 |  | 5 |
|  | 60000 - 80000 |  | 4 |
|  | 40000 – 60000 |  | 3 |
|  | 15000 – 40000 |  | 2 |
|  | < = 15000 |  | 1 |
| Population Density |  | 25 |  |
|  | >50000 |  | 5 |
|  | 25000 - 50000 |  | 4 |
|  | 10000 – 25000 |  | 3 |
|  | 1000 - 10000 |  | 2 |
|  | <1000 |  | 1 |
| Land Use |  | 50 |  |
|  | Built-up |  | 5 |
|  | Non Built-up |  | 3 |

T**able AT4.** Interferograms generated for the study area with details of master image, slave image, perpendicular baseline ($B_{P}$), temporal baseline${(B}_{T})$, and the processing phase. To overcome the effect of temporal decorrelation, the work is divided into 6 phases. Here, P1A, P2A, P3A represents phase-1, phase-2, and phase-3 in ascending direction and P1D, P2D, P3D denotes phase-1, phase-2, and phase-3 in descending direction.

| S.No | Master Date | Slave date | \|  \| \| --- \| |  | Phase | S.No | Master Date | Slave date | \|  \| \| --- \| |  | Phase |
| --- | --- | --- | --- | --- | --- | --- | --- | --- | --- | --- | --- | --- | --- |
| 1 | 13-Dec-15 | 24-Nov-14 | 6 | -384  $B_{T}$(days) | P1A | 36 | 28-Aug-17 | 18-Apr-17 | -57 | -132  $B_{T}$(days) | P2A |
| 2 | 13-Dec-15 | 18-Dec-14 | -14 | -360 | P1A | 37 | 28-Aug-17 | 30-Apr-17 | 8 | -120 | P2A |
| 3 | 13-Dec-15 | 11-Jan-15 | -55 | -336 | P1A | 38 | 28-Aug-17 | 12-May-17 | 2  $B_{P}$(m) | -108 | P2A |
| 4 | 13-Dec-15 | 04-Feb-15 | 69  $B_{P}$(m) | -312 | P1A | 39 | 28-Aug-17 | 24-May-17 | 99 | -96 | P2A |
| 5 | 13-Dec-15 | 12-Mar-15 | 9 | -276 | P1A | 40 | 28-Aug-17 | 05-Jun-17 | -3 | -84 | P2A |
| 6 | 13-Dec-15 | 05-Apr-15 | 72 | -252 | P1A | 41 | 28-Aug-17 | 29-Jun-17 | 19 | -60 | P2A |
| 7 | 13-Dec-15 | 29-Apr-15 | -45 | -228 | P1A | 42 | 28-Aug-17 | 11-Jul-17 | 67 | -48 | P2A |
| 8 | 13-Dec-15 | 16-Jun-15 | 38 | -180 | P1A | 43 | 28-Aug-17 | 23-Jul-17 | 84 | -36 | P2A |
| 9 | 13-Dec-15 | 10-Jul-15 | 45 | -156 | P1A | 44 | 28-Aug-17 | 04-Aug-17 | 24 | -24 | P2A |
| 10 | 13-Dec-15 | 27-Aug-15 | 11 | -108 | P1A | 45 | 28-Aug-17 | 16-Aug-17 | -44 | -12 | P2A |
| 11 | 13-Dec-15 | 02-Oct-15 | -10 | -72 | P1A | 46 | 28-Aug-17 | 09-Sep-17 | 54 | 12 | P2A |
| 12 | 13-Dec-15 | 19-Nov-15 | -23 | -24 | P1A | 47 | 28-Aug-17 | 03-Oct-17 | 32 | 36 | P2A |
| 13 | 13-Dec-15 | 30-Jan-16 | 51 | 48 | P1A | 48 | 28-Aug-17 | 15-Oct-17 | -17 | 48 | P2A |
| 14 | 13-Dec-15 | 23-Feb-16 | 21 | 72 | P1A | 49 | 28-Aug-17 | 27-Oct-17 | -81 | 60 | P2A |
| 15 | 13-Dec-15 | 18-Mar-16 | -9 | 96 | P1A | 50 | 28-Aug-17 | 08-Nov-17 | 14 | 72 | P2A |
| 16 | 13-Dec-15 | 11-Apr-16 | -28 | 120 | P1A | 51 | 28-Aug-17 | 20-Nov-17 | 2 | 84 | P2A |
| 17 | 13-Dec-15 | 05-May-16 | 52 | 144 | P1A | 52 | 28-Aug-17 | 14-Dec-17 | 60 | 108 | P2A |
| 18 | 13-Dec-15 | 29-May-16 | -3 | 168 | P1A | 53 | 28-Aug-17 | 26-Dec-17 | 42 | 120 | P2A |
| 19 | 13-Dec-15 | 16-Jul-16 | 56 | 216 | P1A | 54 | 28-Aug-17 | 07-Jan-18 | -11 | 132 | P2A |
| 20 | 13-Dec-15 | 09-Aug-16 | 14 | 240 | P1A | 55 | 28-Aug-17 | 19-Jan-18 | 1 | 144 | P2A |
| 21 | 13-Dec-15 | 02-Sep-16 | 160 | 264 | P1A | 56 | 28-Aug-17 | 12-Feb-18 | 6 | 168 | P2A |
| 22 | 13-Dec-15 | 26-Sep-16 | -7 | 288 | P1A | 57 | 28-Aug-17 | 24-Feb-18 | -14 | 180 | P2A |
| 23 | 13-Dec-15 | 20-Oct-16 | 21 | 312 | P1A | 58 | 28-Aug-17 | 08-Mar-18 | 30 | 192 | P2A |
| 24 | 13-Dec-15 | 13-Nov-16 | 46 | 336 | P1A | 59 | 28-Aug-17 | 20-Mar-18 | 43 | 204 | P2A |
| 25 | 13-Dec-15 | 07-Dec-16 | -50 | 360 | P1A | 60 | 28-Aug-17 | 01-Apr-18 | 45 | 216 | P2A |
| 26 | 13-Dec-15 | 31-Dec-16 | -18 | 384 | P1A | 61 | 28-Aug-17 | 25-Apr-18 | 30 | 240 | P2A |
| 27 | 28-Aug-17 | 20-Oct-16 | 15 | -312 | P2A | 62 | 28-Aug-17 | 07-May-18 | 21 | 252 | P2A |
| 28 | 28-Aug-17 | 07-Dec-16 | -53 | -264 | P2A | 63 | 28-Aug-17 | 31-May-18 | 93 | 276 | P2A |
| 29 | 28-Aug-17 | 24-Jan-17 | 12 | -216 | P2A | 64 | 28-Aug-17 | 12-Jun-18 | 28 | 288 | P2A |
| 30 | 28-Aug-17 | 05-Feb-17 | -2 | -204 | P2A | 65 | 28-Aug-17 | 24-Jun-18 | 44 | 300 | P2A |
| 31 | 28-Aug-17 | 17-Feb-17 | -3 | -192 | P2A | 66 | 28-Aug-17 | 30-Jul-18 | 78 | 336 | P2A |
| 32 | 28-Aug-17 | 01-Mar-17 | 60 | -180 | P2A | 67 | 28-Aug-17 | 11-Aug-18 | 94 | 348 | P2A |
| 33 | 28-Aug-17 | 13-Mar-17 | -4 | -168 | P2A | 68 | 28-Aug-17 | 16-Sep-18 | -15 | 384 | P2A |
| 34 | 28-Aug-17 | 25-Mar-17 | 26 | -156 | P2A | 69 | 28-Aug-17 | 10-Oct-18 | 52 | 408 | P2A |
| 35 | 28-Aug-17 | 06-Apr-17 | -34 | -144 | P2A | 70 | 14-Jan-19 | 23-Aug-18 | 73 | -144 | P3A |

Table A.1 contd…

| S.No | Master Date | Slave date | $B_{P}$(m)   \|  \| \| --- \| | $B_{T}$(days)   \|  \| \| --- \| | Phase | S.No | Master Date | Slave date | $B_{P}$(m)   \|  \| \| --- \| | $B_{T}$(days)   \|  \| \| --- \| | Phase |
| --- | --- | --- | --- | --- | --- | --- | --- | --- | --- | --- | --- | --- | --- | --- | --- |
| 71 | 14-Jan-19 | 04-Sep-18 | -49 | -132 | P3A | 108 | 19-Feb-16 | 25-Apr-15 | -127 | -300 | P1D |
| 72 | 14-Jan-19 | 16-Sep-18 | 9 | -120 | P3A | 109 | 19-Feb-16 | 19-May-15 | -10 | -276 | P1D |
| 73 | 14-Jan-19 | 10-Oct-18 | 78 | -96 | P3A | 110 | 19-Feb-16 | 12-Jun-15 | 10 | -252 | P1D |
| 74 | 14-Jan-19 | 22-Oct-18 | 78 | -84 | P3A | 111 | 19-Feb-16 | 16-Sep-15 | -16 | -156 | P1D |
| 75 | 14-Jan-19 | 03-Nov-18 | 30 | -72 | P3A | 112 | 19-Feb-16 | 15-Nov-15 | 10 | -96 | P1D |
| 76 | 14-Jan-19 | 15-Nov-18 | -40 | -60 | P3A | 113 | 19-Feb-16 | 09-Dec-15 | 53 | -72 | P1D |
| 77 | 14-Jan-19 | 27-Nov-18 | 48 | -48 | P3A | 114 | 19-Feb-16 | 02-Jan-16 | -11 | -48 | P1D |
| 78 | 14-Jan-19 | 09-Dec-18 | 60 | -36 | P3A | 115 | 19-Feb-16 | 26-Jan-16 | -48 | -24 | P1D |
| 79 | 14-Jan-19 | 21-Dec-18 | 104 | -24 | P3A | 116 | 19-Feb-16 | 02-Mar-16 | -29 | 12 | P1D |
| 80 | 14-Jan-19 | 02-Jan-19 | 63 | -12 | P3A | 117 | 19-Feb-16 | 14-Mar-16 | -50 | 24 | P1D |
| 81 | 14-Jan-19 | 26-Jan-19 | -26 | 12 | P3A | 118 | 19-Feb-16 | 07-Apr-16 | 48 | 48 | P1D |
| 82 | 14-Jan-19 | 19-Feb-19 | 45 | 36 | P3A | 119 | 19-Feb-16 | 01-May-16 | -9 | 72 | P1D |
| 83 | 14-Jan-19 | 03-Mar-19 | -27 | 48 | P3A | 120 | 19-Feb-16 | 25-May-16 | -1 | 96 | P1D |
| 84 | 14-Jan-19 | 15-Mar-19 | -6 | 60 | P3A | 121 | 19-Feb-16 | 05-Aug-16 | 97 | 168 | P1D |
| 85 | 14-Jan-19 | 27-Mar-19 | -56 | 72 | P3A | 122 | 19-Feb-16 | 29-Aug-16 | -57 | 192 | P1D |
| 86 | 14-Jan-19 | 08-Apr-19 | 26 | 84 | P3A | 123 | 19-Feb-16 | 28-Sep-16 | 19 | 222 | P1D |
| 87 | 14-Jan-19 | 14-May-19 | 27 | 120 | P3A | 124 | 19-Feb-16 | 04-Oct-16 | 32 | 228 | P1D |
| 88 | 14-Jan-19 | 26-May-19 | 23 | 132 | P3A | 125 | 19-Feb-16 | 10-Oct-16 | 7 | 234 | P1D |
| 89 | 14-Jan-19 | 19-Jun-19 | 109 | 156 | P3A | 126 | 19-Feb-16 | 16-Oct-16 | -8 | 240 | P1D |
| 90 | 14-Jan-19 | 01-Jul-19 | 77 | 168 | P3A | 127 | 19-Feb-16 | 28-Oct-16 | 18 | 252 | P1D |
| 91 | 14-Jan-19 | 13-Jul-19 | 73 | 180 | P3A | 128 | 19-Feb-16 | 03-Nov-16 | 32 | 258 | P1D |
| 92 | 14-Jan-19 | 25-Jul-19 | -6 | 192 | P3A | 129 | 19-Feb-16 | 09-Nov-16 | -19 | 264 | P1D |
| 93 | 14-Jan-19 | 06-Aug-19 | -16 | 204 | P3A | 130 | 19-Feb-16 | 21-Nov-16 | -6 | 276 | P1D |
| 94 | 14-Jan-19 | 18-Aug-19 | 29 | 216 | P3A | 131 | 19-Feb-16 | 27-Nov-16 | -30 | 282 | P1D |
| 95 | 14-Jan-19 | 30-Aug-19 | 124 | 228 | P3A | 132 | 19-Feb-16 | 03-Dec-16 | 44 | 288 | P1D |
| 96 | 14-Jan-19 | 11-Sep-19 | 68 | 240 | P3A | 133 | 19-Feb-16 | 15-Dec-16 | 30 | 300 | P1D |
| 97 | 14-Jan-19 | 23-Sep-19 | -38 | 252 | P3A | 134 | 19-Feb-16 | 21-Dec-16 | -4 | 306 | P1D |
| 98 | 14-Jan-19 | 05-Oct-19 | -68 | 264 | P3A | 135 | 19-Feb-16 | 27-Dec-16 | 25 | 312 | P1D |
| 99 | 14-Jan-19 | 10-Nov-19 | 32 | 300 | P3A | 136 | 29-Sep-17 | 29-Aug-16 | -39 | -396 | P2D |
| 100 | 14-Jan-19 | 16-Dec-19 | -13 | 336 | P3A | 137 | 29-Sep-17 | 22-Sep-16 | 0 | -372 | P2D |
| 101 | 14-Jan-19 | 28-Dec-19 | 23 | 348 | P3A | 138 | 29-Sep-17 | 28-Sep-16 | 37 | -366 | P2D |
| 102 | 19-Feb-16 | 15-Oct-14 | -41 | -492 | P1D | 139 | 29-Sep-17 | 04-Oct-16 | 50 | -360 | P2D |
| 103 | 19-Feb-16 | 02-Dec-14 | 25 | -444 | P1D | 140 | 29-Sep-17 | 10-Oct-16 | 24 | -354 | P2D |
| 104 | 19-Feb-16 | 26-Dec-14 | 1 | -420 | P1D | 141 | 29-Sep-17 | 16-Oct-16 | 16 | -348 | P2D |
| 105 | 19-Feb-16 | 19-Jan-15 | 6 | -396 | P1D | 142 | 29-Sep-17 | 28-Oct-16 | 35 | -336 | P2D |
| 106 | 19-Feb-16 | 12-Feb-15 | -250 | -372 | P1D | 143 | 29-Sep-17 | 03-Nov-16 | 49 | -330 | P2D |
| 107 | 19-Feb-16 | 01-Apr-15 | -15 | -324 | P1D | 144 | 29-Sep-17 | 09-Nov-16 | 1 | -324 | P2D |

Table A.1 contd…

| S.No | Master Date | Slave date | $B_{P}$(m)  $B_{T}$(days)   \|  \| \| --- \| |  | Phase | S.No | Master Date | Slave date | $B_{P}$(m)  $B_{T}$(days)   \|  \| \| --- \| |  | Phase |
| --- | --- | --- | --- | --- | --- | --- | --- | --- | --- | --- | --- | --- | --- |
| 145 | 29-Sep-17 | 21-Nov-16 | 8 | -312 | P2D | 182 | 29-Sep-17 | 08-Jun-18 | -48 | 252 | P2D |
| 146 | 29-Sep-17 | 27-Nov-16 | -12 | -306 | P2D | 183 | 29-Sep-17 | 20-Jun-18 | -39 | 264 | P2D |
| 147 | 29-Sep-17 | 03-Dec-16 | 61 | -300 | P2D | 184 | 29-Sep-17 | 02-Jul-18 | -36 | 276 | P2D |
| 148 | 29-Sep-17 | 15-Dec-16 | 47 | -288 | P2D | 185 | 29-Sep-17 | 14-Jul-18 | 21 | 288 | P2D |
| 149 | 29-Sep-17 | 21-Dec-16 | 11 | -282 | P2D | 186 | 29-Sep-17 | 26-Jul-18 | 1 | 300 | P2D |
| 150 | 29-Sep-17 | 08-Jan-17 | -45 | -264 | P2D | 187 | 29-Sep-17 | 07-Aug-18 | -9 | 312 | P2D |
| 151 | 29-Sep-17 | 14-Jan-17 | 86 | -258 | P2D | 188 | 29-Sep-17 | 19-Aug-18 | -20 | 324 | P2D |
| 152 | 29-Sep-17 | 20-Jan-17 | -87 | -252 | P2D | 189 | 29-Sep-17 | 31-Aug-18 | 51 | 336 | P2D |
| 153 | 29-Sep-17 | 01-Feb-17 | -16 | -240 | P2D | 190 | 29-Sep-17 | 12-Sep-18 | 0 | 348 | P2D |
| 154 | 29-Sep-17 | 07-Feb-17 | -25 | -234 | P2D | 191 | 14-Aug-19 | 31-Aug-18 | -10 | -348 | P3D |
| 155 | 29-Sep-17 | 13-Feb-17 | 66 | -228 | P2D | 192 | 14-Aug-19 | 12-Sep-18 | -57 | -336 | P3D |
| 156 | 29-Sep-17 | 09-Mar-17 | -27 | -204 | P2D | 193 | 14-Aug-19 | 24-Sep-18 | -47 | -324 | P3D |
| 157 | 29-Sep-17 | 21-Mar-17 | -48 | -192 | P2D | 194 | 14-Aug-19 | 06-Oct-18 | -111 | -312 | P3D |
| 158 | 29-Sep-17 | 02-Apr-17 | 7 | -180 | P2D | 195 | 14-Aug-19 | 18-Oct-18 | -50 | -300 | P3D |
| 159 | 29-Sep-17 | 08-May-17 | 98 | -144 | P2D | 196 | 14-Aug-19 | 30-Oct-18 | -48 | -288 | P3D |
| 160 | 29-Sep-17 | 20-May-17 | 32 | -132 | P2D | 197 | 14-Aug-19 | 11-Nov-18 | -14 | -276 | P3D |
| 161 | 29-Sep-17 | 01-Jun-17 | -79 | -120 | P2D | 198 | 14-Aug-19 | 05-Dec-18 | -2 | -252 | P3D |
| 162 | 29-Sep-17 | 13-Jun-17 | 28 | -108 | P2D | 199 | 14-Aug-19 | 17-Dec-18 | -161 | -240 | P3D |
| 163 | 29-Sep-17 | 25-Jun-17 | 65 | -96 | P2D | 200 | 14-Aug-19 | 29-Dec-18 | -61 | -228 | P3D |
| 164 | 29-Sep-17 | 31-Jul-17 | 29 | -60 | P2D | 201 | 14-Aug-19 | 10-Jan-19 | -76 | -216 | P3D |
| 165 | 29-Sep-17 | 12-Aug-17 | 28 | -48 | P2D | 202 | 14-Aug-19 | 22-Jan-19 | 17 | -204 | P3D |
| 166 | 29-Sep-17 | 24-Aug-17 | 20 | -36 | P2D | 203 | 14-Aug-19 | 03-Feb-19 | 14 | -192 | P3D |
| 167 | 29-Sep-17 | 05-Sep-17 | 19 | -24 | P2D | 204 | 14-Aug-19 | 27-Feb-19 | -41 | -168 | P3D |
| 168 | 29-Sep-17 | 17-Sep-17 | 44 | -12 | P2D | 205 | 14-Aug-19 | 23-Mar-19 | 45 | -144 | P3D |
| 169 | 29-Sep-17 | 23-Oct-17 | 37 | 24 | P2D | 206 | 14-Aug-19 | 28-Apr-19 | 12 | -108 | P3D |
| 170 | 29-Sep-17 | 04-Nov-17 | 49 | 36 | P2D | 207 | 14-Aug-19 | 03-Jun-19 | 22 | -72 | P3D |
| 171 | 29-Sep-17 | 28-Nov-17 | -30 | 60 | P2D | 208 | 14-Aug-19 | 26-Aug-19 | -33 | 12 | P3D |
| 172 | 29-Sep-17 | 10-Dec-17 | -70 | 72 | P2D | 209 | 14-Aug-19 | 07-Sep-19 | -57 | 24 | P3D |
| 173 | 29-Sep-17 | 15-Jan-18 | 32 | 108 | P2D | 210 | 14-Aug-19 | 19-Sep-19 | -91 | 36 | P3D |
| 174 | 29-Sep-17 | 27-Jan-18 | -55 | 120 | P2D | 211 | 14-Aug-19 | 01-Oct-19 | 35 | 48 | P3D |
| 175 | 29-Sep-17 | 08-Feb-18 | -8 | 132 | P2D | 212 | 14-Aug-19 | 19-Oct-19 | -49 | 66 | P3D |
| 176 | 29-Sep-17 | 20-Feb-18 | 8 | 144 | P2D | 213 | 14-Aug-19 | 31-Oct-19 | -117 | 78 | P3D |
| 177 | 29-Sep-17 | 04-Mar-18 | 43 | 156 | P2D | 214 | 14-Aug-19 | 12-Nov-19 | -38 | 90 | P3D |
| 178 | 29-Sep-17 | 09-Apr-18 | -27 | 192 | P2D | 215 | 14-Aug-19 | 24-Nov-19 | -47 | 102 | P3D |
| 179 | 29-Sep-17 | 21-Apr-18 | -88 | 204 | P2D | 216 | 14-Aug-19 | 06-Dec-19 | -100 | 114 | P3D |
| 180 | 29-Sep-17 | 03-May-18 | -12 | 216 | P2D | 217 | 14-Aug-19 | 18-Dec-19 | -55 | 126 | P3D |
| 181 | 29-Sep-17 | 27-May-18 | 27 | 240 | P2D | 218 | 14-Aug-19 | 30-Dec-19 | -6 | 138 | P3D |
|  | | | | | | 219 | 14-Aug-19 | 05-Jan-20 | -105 | 144 | P3D |
